# Digital Anthropometry: A Systematic Review on Precision, Reliability and Accuracy of Most Popular Existing Technologies

**DOI:** 10.3390/nu15020302

**Published:** 2023-01-07

**Authors:** Edoardo Mocini, Camillo Cammarota, Francesco Frigerio, Luca Muzzioli, Claudia Piciocchi, Doriana Lacalaprice, Fabio Buccolini, Lorenzo Maria Donini, Alessandro Pinto

**Affiliations:** 1 Department of Experimental Medicine, Sapienza University, 00185 Rome, Italy; 2 Department of Mathematics, Sapienza University, 00185 Rome, Italy; 3 3C Engineering Srls, 00177 Rome, Italy

**Keywords:** digital anthropometry, 3D body imaging, body scanning, anthropometry, body composition, systematic review

## Abstract

Digital anthropometry (DA) has been recently developed for body composition evaluation and for postural analysis. The aims of this review are to examine the current state of DA technology, as well as to verify the methods for identifying the best technology to be used in the field of DA by evaluating the reliability and accuracy of the available technologies on the market, and lay the groundwork for future technological developments. A literature search was performed and 28 studies met the inclusion criteria. The reliability and accuracy of DA was high in most studies, especially in the assessment of patients with obesity, although they varied according to the technology used; a good correlation was found between DA and conventional anthropometry (CA) and body composition estimates. DA is less time-consuming and less expensive and could be used as a screening tool before more expensive imaging techniques or as an alternative to other less affordable techniques. At present, DA could be useful in clinical practice, but the heterogeneity of the available studies (different devices used, laser technologies, population examined, etc.) necessitates caution in the interpretation of the obtained results. Furthermore, the need to develop integrated technologies for analyzing body composition according to multi-compartmental models is increasingly evident.

## 1. Introduction

Conventional human anthropometry is a simple, non-invasive, and economical methodology that is easy to perform in different epidemiological or clinical settings and aims to collect measurements of the human body at a total body and/or regional level using simple devices (stadiometer, weight scale, meter, gauges, compasses, skinfold caliper, etc.) [1]. In a broader perspective, it is correct to consider anthropometry as the measurement of each segment, area, or volume of the human body.

The etymology of the word anthropometry is based on the Greek anthropos (human) and metron (measure). Anthropometry was developed in the late 19th century by anthropologists analyzing the differences in human body shape. The role of anthropometry in evaluating nutritional status was defined at the end of the 19th century by Richer, who used the thickness of skin folds as an index of fatness. The modern era of nutritional anthropometry began with Matiegka’s studies during the First World War [2]. Matiegka’s interest in the physical efficiency of soldiers led him to develop methods for anthropometrically subdividing the human body into muscle, fat, and bone compartments.

Anthropometry’s main limitation is that it is deeply dependent on the operator’s skill and requires adequate training. The International Society for the Advancement of Kinanthropometry (ISAK) has established a methodology for reducing procedural errors [3]. Kinanthropometry, first defined by William Ross in 1978, is the study of body size, shape, proportions, composition, and function in order to understand growth process, functional and sporting performances, and nutritional status [4]. Sometimes the two terms, anthropometry and kinanthropometry, are used interchangeably, even if they have different meanings. Anthropometry is based on simple body measures such as weight, height, sitting height, lengths and diameters of the different body segments, circumferences, skinfold thickness, etc., and some parameters are included in predictive equations to estimate indirectly the body composition or used as direct indicators of disease risk (i.e., waist circumference, sagittal abdominal diameter).

Notwithstanding the procedure standardization promoted by existing guidelines, human anthropometry is limited by precision and accuracy, undermining its applicability, particularly at the individual level. Anthropometric measurement errors, categorized as random errors (expressed in terms of precision and reliability) and systematic errors (expressed in terms of accuracy), are described in Box 1 and Box 2.

Box 1 **Precision, reliability, accuracy, validity [5].**

  Confusion often arises when applying these terms to the field of anthropometry, leading some authors to use them interchangeably. Following are practical definitions.

  **Precision**: it expresses how similar two repeated measurements are to each other, under similar conditions (i.e., how results can be duplicated from one measurement to another). It can be intra-rater (duplicate measurements on the same subject by a single rater) or inter-rater (two raters measuring the same variable on the same subject).

  **Reliability**: it shares the same features as precision, but also includes individual differences due to dependability or physiological variation of the measured variable (independent of technique errors). For example, within each individual’s height there is intra-day variation due to changes in intervertebral disk hydration.

  Precision and reliability are sometimes used interchangeably, but low precision can coexist with high reliability (with a wider range of measurement values, e.g., when measurements are made on a heterogeneous group of individuals) and vice versa.

  **Accuracy**: it expresses how close a measurement is to the “true value” of the evaluated variable. For example, measurements by a field technique can be compared with those by a gold standard method: the higher the accuracy, the closer their respective values. Similarly, measurements by an unexperienced anthropometrist can be compared with those by an expert/reference measurer.

  **Validity**: it expresses the extent to which a study evaluates what it was intended to evaluate. Results from a clinical study are deemed internally valid if they are not biased by study design flaws and externally valid if they can be generalized to the population of reference. In psychometry, validity expresses the degree to which a scale/test measures the construct it was developed for in the first place. A test cannot be considered valid/invalid in itself without taking into account the subjects and context it is applied to.

  Accuracy and validity are used interchangeably by some authors.

Box 2 **Random and systematic errors in anthropometric measures [3,6,7,8,9,10].**

- **Random error** can be expressed in terms of **precision** and **reliability (relative or absolute)** level.
  - **Precision** expresses the variability between repeated measurements by a particular observer using a particular device to measure a particular variable. Imprecision can be caused by flawed measuring equipment, inadequately trained measurers, or poor technique. Common indices of precision are absolute intra- and inter- Technical Error of Measurement (TEM) and relative TEM (%TEM). According to the International Society for the Advancement of Kinanthropometry (ISAK) protocol, acceptable TEMs are 0.1 kg, 3 cm and 2 cm for weight, stature, and body circumferences, respectively. Another example is the precision error of repeated measurements (PE).
  - **Absolute reliability** regards the consistency of scores for individuals or, in other terms, the degree to which repeated measurements vary for individuals. It can be expressed by the coefficient of variation (CV) and the standard error of measurement of a group estimate (SEM).
  - **Relative reliability** is the degree to which individuals maintain their position in a sample over repeated measurements; it is expressed by the reliability coefficient R and the intraclass correlation coefficient (ICC).
- **Systematic error or bias** depends on accuracy, defining the level of correlation or agreement between an under-validation (bedside) method and a reference method when measuring the same variable. It may depend on equipment bias (lack of calibration, device complexity) or operator error. As mentioned before, it is possible to classify accuracy in terms of:
  - **Correlation at a mean level**: paired *t*-tests, Pearson’s correlation coefficient, concordance correlation coefficient (CCC), linear regression. The latter involves calculating the coefficient of determination (R^2^), standard error of the estimate (SEE), and root mean square error (RMSE). CCC appears useful to describe methods agreement (association and identity) when more than two operators and/or repeated measurements come into play. While ICC relies on ANOVA assumptions, CCC does not; both indices concurrently involve precision and accuracy assessment.
  - **Agreement or concordance, at an individual level**: the Bland–Altman plot compares the level of agreement of two different technologies by plotting the difference against the arithmetic mean (M) of each pair of measurements, the total mean difference (bias), and the 95% limits of agreement (LoA), with their respective confidence intervals.

**Random error**
  - Precision
    ○ Absolute Technical Error of Measurement (TEM) = √(ΣD^2^/2*n*)
    ○ Relative Technical Error of Measurement (%TEM) = (TEM/M) × 100
    ○ Precision Error (PE) = √(ΣSD^2^/*n*)
    D = Δ between the 2 measurements, n = number of subjects, M = arithmetic mean of measurements, SD = standard deviation of measurements
  - Absolute Reliability
    ○ Coefficient of Variation (CV) = SD/M
    ○ Standard Error of Measurement (SEM) = SD√(1 − rxx)
    rxx = chosen coefficient of reliability, usually ICC
  - Relative Reliability
    ○ Reliability Coefficient (R) = 1 − (total TEM^2^/SD^2^)
                    1 − (%TEM/CV)^2^
    ○ Intraclass correlation coefficient (ICC)
      different forms according to the ANOVA model, number of raters, and consistency/absolute agreement
    SD^2^ = total inter-subject variance
**Systematic error or bias**
  - Correlation, at a mean level
    ○ Pearson’s correlation coefficient (r)
    ○ Coefficient of determination (R^2^)
    ○ Standard Error of the Estimate (SEE) = √[Σ(Y − X)^2^/(*n* − 2)]
    ○ Root Mean Square Error (RMSE) = √[Σ(Y − X)^2^/*n*]
    ○ Concordance Correlation Coefficient (CCC)
    ○ Mean difference (groups)
    ○ Paired *t*-tests
    Y = measured value; X = predicted value
  - Agreement or concordance, at an individual level
    ○ Bland–Altman plot
    ○ LoA = mean difference ± (1.96 × SD)
    Y = reference method; X = bedside method

To date, anthropometry refers to the systematic collection of physical measurements of the human body [11] and their combination to develop useful indicators for the assessment of nutritional status [12], the risk of malnutrition, sarcopenia, the decline in physical capacity and performance, the impairment of quality of life, and the increased risk of developing non-communicable diseases (metabolic diseases, diabetes or cardiovascular disease) [13,14]. Anthropometric measures are also included in tools for diagnosing or assessing the risk of malnutrition and monitoring the effectiveness of nutritional intervention strategies. Furthermore, anthropometric parameters are useful for analyzing the relationships between lifestyle (eating habits and level of physical activity) and nutritional status, because, although linked to the genetic heritage, they are also influenced by lifestyle, eating habits, physical activity, and environmental, social, and cultural factors [15,16,17].

During the mid-1980s, a textile manufacturer asked the University of Loughborough, England, to provide a new technology to obtain comprehensive body shape data with the aim of facilitating garment production. The company wanted to explore the possibility of developing a contactless machine that was reasonably portable and fast enough to economically scan a large sample of the British population. In 1987, the Loughborough Anthropometric Shadow Scanner, LASS, was realized. The device included a camera, a projector, and a 360° rotating table on which the volunteer had to be positioned during the evaluation procedure. This technology gave birth to the field of digital anthropometry (DA). Over the next three decades, a rapid advance in methodologies designed to quantify the shape of the human body occurred, including laser and light technologies, millimeter-wave radar, and multi-perspective camera methods. Interest in automated or digital anthropometry has intensified with the introduction of relatively inexpensive optical imaging devices that replace the LASS system camera. Currently, practical three-dimensional (3D) imaging devices are available either for clinical use or for personal use at home.

Recent technological advances have positively influenced the development of 3D imaging, enabling faster and more reliable anthropometric measurements and reducing physical contact between patients and operators. Initially confined to the textile industry, today digital anthropometry plays an important role in the assessment of body composition and postural analysis, e.g., as a first-line screening tool for scoliosis prior to Cobb angle measurement via a conventional X-ray image [1]. Consequently, in the near future, digital anthropometry could help overcome the traditional assumptions and limitations of classical anthropometry.

This systematic review aims, as a first objective, to provide an update on the state of digital anthropometry in medicine, with specific reference to body circumferences and lengths, body shape.

In the context of bicompartmental models of body composition that distinguish Fat Mass (FM) and Fat Free Mass (FFM), digital anthropometry allows the estimation of the body volume which, knowing the mass (weight), can be used to calculate the body density. Starting from the body density, through the use of predictive equations validated on samples of individuals differentiated by ethnicity, sex, and age, it is possible to estimate the body composition (FM%).

Starting from the analysis of existing technologies, a secondary objective, is to identify the best technologies to be used in the field of DA or which technologies are most suitable for different fields of application.

The review will cover not only the precision, reliability, and accuracy of existing technologies, but also the cost/benefit assessments related to the collection of anthropometric and body composition measurements for the diagnosis and treatment of diseases in different clinical settings.

## 2. Materials and Methods

This systematic review was carried out according to the Preferred Reporting Items for Systematic Reviews and Meta-Analyses (PRISMA) statement [18].

### 2.1. Search Strategy

The bibliographic search was carried out using three different electronic databases, PubMed, Embase, and Scopus, without any restrictions on the age of the recruited population. The year of publication of studies was restricted to 2000–present (i.e., September, 2021). The research was conducted by applying the PICO methodology (Population: healthy population; Intervention: use of DA; Comparator: use of conventional techniques for the generation of anthropometric data; Outcome: replacement of conventional techniques with DA). The last search was performed on 15 September 2021.

The following free-text keywords were searched: “Digital Anthropometry”, “Anthropometry Digital Human Model”, “Digital Human Model Anthropometry”, “Digital Body Size”, “Digital Body Shape”, “Body Scanners”, “Body Scanning”, “Body Optical Imaging”, “Digital Anatomic measurements”, “Optical scan to anthropometric data generation”, “Optical imaging technology for body size”, “Optical imaging technology for body shape”, “Whole body surface scanners”, “Digital Anthropometrics system”.

### 2.2. Eligibility Criteria and Procedures for Article Selection

The included studies involved the use of DA to analyze lengths, circumferences, and other anthropometric measurements. Body composition results were also included because of the close connection, through the use of predictive equations, that exists in numerous technologies, including reference methods, between anthropometric measurement and body composition appraisal.

For these reasons we decided to include studies concerning body composition, considering them an indirect measure of the precision and accuracy of the technologies used to measure anthropometric measurements that are then used to estimate body composition.

Each selected study compared DA with conventional techniques of body composition assessment, such as manual measurements, bioimpedance analysis (BIA), dual-energy X-ray absorptiometry (DXA), hydrostatic weighing (HW), air displacement plethysmography (ADP) and computed tomography (TC). Every study included in this review evaluated accuracy; some studies evaluated both precision and accuracy; studies evaluating only precision but not accuracy were not included.

Meta-analyses, reviews, book chapters, case reports/series, expert opinions, articles in languages other than English, full text unavailable, articles published before 2000, and those concerning somatotypes, body typing with statistical models, body surface, and postural analysis were discarded.

The references of included studies were also checked to identify other potentially relevant studies. The search process was carried out by two researchers (F.F. and P.C.) working independently; disagreements were solved through consensus and by discussion with the lead author (M.E.).

### 2.3. Data Extraction and Quality Assessment

Data was extracted by the lead author, who retrieved the following information for each study: first author, year of publication, study design, country of origin, type of 3D scanner used, comparison with other techniques, and results.

The methodological quality of the included studies was assessed by means of the Appraisal tool for Cross-Sectional Studies (AXIS) [19] and the Newcastle–Ottawa Scale (NOS) on cross-sectional studies [20]. Scores for both scales are reported at the end of this section.

The AXIS tool consists of 20 items that evaluate the quality of reporting (7 questions), study design quality (7 questions), and potential introduction of biases in a study (6 questions). Each question has three possible answers: “yes”, “no”, “do not know/comment”, therefore implying a subjective judgement by the user. While numerical rating scales may appear to provide a more objective assessment, increasing comparability among different raters, in reality summing up individual item answers to produce a global score or a weighted summarization (as in a meta-analysis) can lead to biased estimates since quality itself can be non-additive and nonlinear [21]. On the other hand, the AXIS provides more flexibility for quality of reporting and risk of bias assessment, due to its inherent subjectivity; it appears more comprehensive than similar tools for cross-sectional studies [19].

The NOS, appropriately modified for cross-sectional studies assessment, consists of seven different items grouped into three categories; each item is given a score ranging from 0 to (maximum) 2 stars by the rater. A summary score ranging from 0 to 10 stars is computed by adding up the individual item scores. The three categories are: quality of group selection (4 questions, maximum 5 stars overall), comparability between groups (1 question, maximum 1 star overall), and study outcomes (2 questions, maximum 3 stars overall). The NOS summary score has no universal cut-off value but some authors have suggested the following categorization: very good (9–10 stars), good (7–8 stars), satisfactory (5–6 stars), unsatisfactory (0–4 stars) [22].

Finally, the quality of evidence and strength of recommendations were evaluated using the GRADE scale (Grading of Recommendations Assessment, Development, and Evaluation). The GRADE approach gives an a priori ranking based on study design (randomized controlled trial or observational study) before grading certainty of evidence and weighing cost-effectiveness, patient preference and desiderable/undesiderable effects balance [23].

## 3. Results

The search returned 4410 references: a total of 696 records were found from the search in PubMed, 232 in Embase, 3289 in Scopus, and 194 from the references of some studies. 804 records were excluded because they were duplicates. Another 3519 articles were excluded after screening the remaining citations based on title and abstract. Full-text examination was then conducted, and finally 28 papers were included in this review.

**Figure 1:**
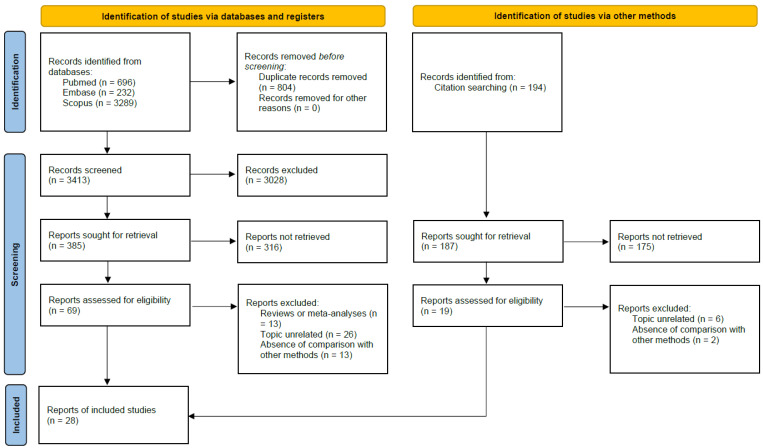
PRISMA 2020 Flowchart From: [18].

A flowchart of the paper selection process is shown in Figure 1. The detailed PRISMA checklist is available in Supplementary Materials Table S1.

The main characteristics of the included studies are summarized in Table 1: all the studies are cross-sectional and they were published between 2006 and 2021. The 4693 participants in the 28 studies have an average age of 27.8 ± 12.6 years (range 2–83). Some studies evaluated only male subjects [24,25,26,27], while others evaluated only female subjects [24,25,26,27,28,29,30,31,32], but both sexes are considered equally: 52% of participants were females, while 48% were males. Regarding the country of origin, nineteen studies were published in the USA [26,29,31,32,33,34,35,36,37,38,39,40,41,42,43,44,45,46,47], three in Switzerland [24,25,27], one in the United Kingdom [48], two in Slovenia [49,50], one in Malaysia [28], one in Italy [30] and one in China [51].

### 3.1. Quality Assessment

The results of the quality assessment of the included studies are available in Supplementary Materials Tables S2 and S3.

The quality assessment by the NOS on cross-sectional studies [20] and by the AXIS tool [19] was conducted by two authors (P.C. and F.F.) independently, and disagreements were resolved by consensus in the presence of a third author (M.E.).

The mean value obtained using the NOS on the cross-sectional studies was 5.2 ± 0.5. The highest value was 6/10 and the minimum value was 4/10. Only eight studies scored above the mean value; considering the three categories, the quality of group selection was assessed as medium risk of bias, the comparability between the groups had a minimum score (high risk of bias), and the study outcome had a maximum score (low risk of bias). Overall, the quality of the studies can be regarded as satisfactory.

Applying the AXIS tool, questions on non-responders (n.7, n.13, n.14) received the “do not know/comment” answer across all studies, since no information on non-responders was available in the included studies. The overall quality score of the studies was fourteen out of twenty. Nine studies had a quality score of sixteen [26,38,40,41,42,43,44,45,47], eight studies of fifteen [24,25,29,31,32,34,36,39], three studies of fourteen [30,37,48], four studies of thirteen [27,33,35,50], one study of twelve [46], two studies of eleven [28,49], and one study of nine [51]. The overall quality of the studies included in the review is good.

Finally, the quality of evidence and strength of recommendations were evaluated using the GRADE scale [23].

### 3.2. Comparison between Digital Anthropometry (DA; 3D Scanners) and Classic Manual Anthropometry (CA) Measurements: Body Circumferences, Lengths, and Shape

The results of the studies comparing digital anthropometry (DA; 3D scanners) to classic manual anthropometry (CA) with specific reference to body circumferences, lengths, and shapes, are discussed separately on the basis of reliability and accuracy criteria, which quantify random and systematic (bias) error related to anthropometric measurements, respectively (Box 1 and Box 2). The results of studies included in this systematic review are summarized in Table 2, Table 3, Table 4 and Table 5; the comparison between studies is described in Supplementary Materials Tables S4 and S5.

The measurement results below indicate the minimum value and the maximum value; the ranges are wide due to the differences in values of the measured body section and the laser technology used to measure them.

#### 3.2.1. Reliability

Fourteen out of 30 studies compared the repeatability of digital (DA) and conventional anthropometric (CA) measurements. Almost all studies showed that both methodologies are reliable [24,34,37,38,39,48,50]; however, some studies showed greater reliability for manual measurements than digital scanners [34,37,38,39,48], whereas in other cases the opposite was observed [24,50].

The reliability of the two methodologies (i.e., the variability observed among repeated measurements performed on the same subject by one or more operators, i.e., the intra- and inter-operator variability) is expressed in terms of Technical Error of Measurement (TEM) and %TEM. According to the ISAK protocol, if TEM is <2 cm and %TEM is <1.5%, the anthropometric measurements should be considered reliable.

The calculated TEMs were generally less than 2 cm [38,47,48], with more accurate results observed in manual anthropometry [38,48].

By means of SL-IR (Structured Light-InfraRed) devices, Conkle used the AutoAnthro Scanner^®^ (Occipital San Francisco, CA, USA) to obtain comparable inter- and intra-observer TEM, demonstrating that DA performance was operator independent. In contrast, CA produced higher inter-observer TEMs than intra-observer TEMs, as might be expected [38].

Koepke et al. employed an SL laser scanner (BS VITUS Smart XXL (Human solution GmbH, Kaiserslautern, Germany)), which showed acceptable TEMs for hip circumference, as observed in CA; otherwise, manual measurements of other body sites appeared to be less precise [24].

Tinsley et al. used three different SL-IR scanners (Naked Labs 3D Fitness Trackers^®^ (Redwood City, CA, USA), Fit3D Proscanner^®^ (Redwood City, CA, USA), and Size Stream SS20^®^ (Cary, NC, USA)) and one ToF (Time-of-Flight) device (Styku S100 scanner (Los Angeles, CA, USA)) to compare the repeatability in measuring body circumferences. The four body scanners produced an overall mean root mean-square (RMS)-% CV (% TEM) of 1.1–1.3%, with lower values for hips and waist (%TEM < 1% and 0.7–1.6%, respectively) and higher values for the thigh (0.8–1.4%), neck (1.2–2.0%), and arm circumferences (1.4–2.8%) [47]. Lu used the mean absolute differences (MAD) between the repeated measurements derived from scans, and all MAD values were less than 7 mm [51].

Regarding the relative reliability indices (i.e., the proportion of variance attributable to between-subject variance in a set of measurements), the ICC (two-way mixed-model, absolute agreement) and R (coefficient of reliability) values were high [24,38,47].

Tinsley, Benavides et al. in the aforementioned study, used SL-IR and reported high ICCs (0.974–0.999) for all circumferences [47]; similarly, using SL-IR, Conkle observed high R and ICC values for both methods [38], with slightly better values in CA. Wang et al. showed ICCs > 0.97 for both lengths and circumferences with a SL-laser scanner [46]. Koepke obtained ICCs > 0.993 except for the chest circumference (ICC 0.981) [24]. Pepper et al. reported ICCs ≥ 0.99 for all eight repeated measures of body circumferences, with the abdomen, waist, and hip showing the highest values (ICC = 1.00) and the chest circumference having the lowest one (ICC = 0.992) [31].

For absolute reliability (i.e., consistency within repeated measurements of the same subject), %CV and standard error of the measurement (SEM) were calculated. Wong observed CVs < 5% when using an SL device (Fit3D Proscanner^®^ (Redwood City, CA, USA)) with the exception of the forearm circumference (CV value = 6.09%) [37]; Ng et al. reported %CVs of 0.75–2.24% measured by a SL-IR scanner (Fit3D Proscanner^®^ (Redwood City, CA, USA)) [45]. The studies of Kennedy with SL-IR scanners showed lower reliability in DA than CA for body circumferences (CV 0.4–2.7% vs. 0.2–0.4%), with the most precise measurement being the hip [39]. Bourgeois, who used two SL scanners and one ToF, revealed %CVs < 2.6% for four circumferences: waist, hip, right arm, and right thigh [34]. Simenko and Busic’s studies, which used SL-visible light scanners, reported %CV > 5% [49,50], with the exception of hip circumference (%CV in DA 4.243%, in CA 4.295%) [49], which performed slightly better in DA (%CV 6.62–11.29, SEM 0.13–0.46) [50]. Busic also reported similar values of SEM between the two methods, with higher values in chest and breast circumference [49]. Wong examined the %CV of body shape indices and found that the CV was 1.50% for the waist hip ratio, 1.82% for waist height ratio, and 1.29% for waist-width ratio, respectively [37].

#### 3.2.2. Accuracy

Most of the studies selected evaluated DA accuracy compared with manual measurements. The correlation was studied with Pearson’s coefficient (r), and a very strong linear correlation (r > 0.8) [52] was demonstrated between the two methods [24,27,32,48,49]. There was also a strong correlation with body shape [24]. Some studies [24,27] examined the correlation with Lin’s concordance correlation (CCC); other studies [25] used Spearman Rho, and the strong correlation was confirmed.

Through Pearson’s coefficient and linear regression analysis, it is possible to confirm the goodness of fit with the coefficient of determination (R^2^). In Busic’s study, this value was acceptable: it was over 90% in 7 out of 15 measurements, over 80% in 6 measurements, and above 74.9% in 2 measurements [49]. Bourgeois demonstrated that DA was significantly correlated with CA using the KX-16 scanner^®^ (TC LABS, Apex, NC, USA) (R^2^ 0.71–0.91, *p* < 0.0001; root mean-square error (RMSE), 3.9–7.7), Fit3D Proscanner^®^ (Redwood City, CA, USA) (R^2^ 0.79–0.92, *p* < 0.0001; RMSE, 1.9–6.4) and Styku S100 scanner^®^ (Los Angeles, CA, USA) (R^2^ 0.73–0.96, *p* < 0.0001; RMSE, 2.6–6.3) [34]; similarly, Kennedy and Smith observed that DA significantly predicted manual measurements (R^2^: Fit3D Proscanner^®^ (Redwood City, CA, USA), 0.70–0.96; Styku S100 scanner® (Styku, Los Angeles, CA, USA), 0.54–0.97; Size Stream (Cary, NC, USA), 0.68–0.97; *p* < 0.01) [42]. The explained variance in linear regression by DA was high also in Kennedy’s study (R^2^ 0.84–0.97, *p* < 0.0001), although the Naked Body Scanners (Naked Labs Inc, Redwood City, CA, USA) significantly overestimated waist circumferences by ~2.0 cm compared with reference estimates (*p* < 0.0001). Significant bias was also discovered in measurements of the left and right thighs (*p* < 0.0001). The scanner overestimated thigh circumferences by ~3.0 cm, with significantly greater overestimations for thighs < 55.0 cm [39].

Similar results were found in Sobhyeh and Kennedy’s study (R^2^ > 0.90 for most of the scanners compared with conventional anthropometry, *p* < 0.001, RMSE 1–3 cm), with a few exceptions for limbs; specifically, correlations between CA and DA waist circumference had R^2^s of 0.95–0.97 (*p* < 0.001) [43]. Wong, who studied a pediatric population, found high values of R^2^ and RMSE for waist (R^2^ 0.939, RMSE 3.783 cm) and hip (R^2^ 0.987, RMSE 1.828) circumferences [37]. In the study of Wells, ranking consistency was high (R^2^ > 0.90 for most of the outcomes) [48].

Differences between two methods were found. A statistically significant overestimation between means obtained with DA and CA was observed for waist circumferences (Japar’s study: 85.34 vs. 84.63 cm, *p* < 0.05 [28]; Wells’s study: 83.15 vs. 81.65, *p* < 0.001 [48]; Koepke’s study: 81.38 vs. 80.31 cm, *p* < 0.001 [24]), hip circumferences (Japar’s study: 103.47 vs. 94.88 cm, *p* < 0.01 [28]; Koepke’s study: 99.19 vs. 94.77 cm, *p* < 0.01 [24]), chest circumferences (Koepke’s study: 97.62 vs. 93.82 cm, *p* < 0.001 [24]), and buttock circumferences (Koepke’s study: 97.23 vs. 84.61 cm, *p* < 0.001 [24]). This was also confirmed by Bland–Altman plots that highlighted a systematic bias and a proportional bias in circumference of waist [27]. Wells found a significant bias for the circumferences of the chest (intercept: coefficient −0.88, confidence interval (CI): −0.97 to 0.21; slope: coefficient 1.07, CI 1.05 to 1.08), waist (intercept: coefficient 1.43, CI: 0.34 to 2.51; slope: coefficient 1.00, CI 0.98 to 1.02), knee (intercept: coefficient 0.17, CI −0.48 to 0.83; slope: coefficient 1.04, CI 1.02 to 1.07), and calf (intercept: coefficient 0.37, CI −0.04 to 0.78; slope: coefficient 1.01, CI 0.99 to 1.02) that varied with outcome size and ethnicity [48]. Wong found a good agreement using Bland–Altman plots for hip and waist circumference [37]. Heuberger also reported significant (*p* < 0.01) differences in waist and hip circumference measured by DA and CA [29].

Underestimation of height with DA compared with CA is shown in studies by Koepke (178.31 vs. 180.32 with *p* < 0.001) 24], Beckmann (177.92 vs. 178.69 with *p* < 0.001) [27], and Sager [25], as shown in Bland–Altman plots [24,25,27]. Height was overestimated in DA compared with CA in Conkle’s study (mean differences: 0.59, limits of agreement (LoA): −0.1 to 1.2) [38]. Conkle studied the head circumference, which was overestimated (mean differences: 0.32, LoA: −0.1 to 0.8), and arm circumference, which was underestimated (mean differences: −0.19, LoA: −0.6 to 0.2) [38].

Kennedy et al. found mean group differences between DA and CA ranging from 1.5 cm (arms) to 3.2 cm (thighs), which were all statistically significant apart from that for hip circumference [39]. Sobhyeh et al. reported statistically significant differences between CA and DA means (absolute mean difference (∆)~2 cm across digital scanners and body sites, with few outliers). Overall, Bland–Altman analyses revealed systematic bias in 11 of the 33 evaluations, with the highest observed slopes comparing CA and DA results by the Fit3D Proscanner^®^ (Redwood City, CA, USA) system. Relative CA-DA differences were smaller for chest, waist, and hip measurements (2–3%) and larger for arms (5–7%) and ankle measurements (8–10%). As for lower limbs, the Styku S100 scanner® (Styku, Los Angeles, CA, USA) displayed absolute mean differences between DA and CA measurements, increasing from 1–2 cm at the thighs to 2–3 cm at the calves and then to 6 cm at the ankles; in contrast, both the Fit3D Proscanner^®^ (Redwood City, CA, USA) and the Size Stream SS20 (Cary, NC, USA) showed relatively constant (1–3 cm) mean differences between DA and corresponding CA measurements at those body sites, with no increasing pattern moving down along the legs. As a result, Bland–Altman slopes comparing ankle circumferences that were measured on the Size Stream SS20 (Cary, NC, USA) and Fit3D Proscanner^®^ (Redwood City, CA, USA) were close to zero. When comparing ankle circumferences measured on either of these scans with Styku S100 scanner® (Styku, Los Angeles, CA, USA) scans, the Bland–Altman slopes were larger [43].

Finally, Ng et al. reported small but significant mean differences for waist and hip circumferences (mean differences of 1.75 cm, CI 0.58–2.91 and 3.17 cm, CI 1.93–4.41, respectively, *p* < 0.05) [45].

As for body shape, Japar and Koepke observed significant differences for Waist to Hip Ratio (WHR). Koepke reported a WHR of 0.82 in DA vs. 0.85 in CA, and Japar reported 0.82 in DA vs. 0.89 in CA; for Waist to Height Ratio (WHtR), Koepke reported a value of 0.46 in DA vs. 0.45 in CA, with *p* < 0.001 [24,28].

Simenko reported conflicting results, with 10 statistically different paired measurements out of 14, but clinically small differences (mean differences 0.273–0.974 cm, *p* < 0.05). Bland–Altman plots showed high agreement between both methods; 95% LoA were narrow for both the upper (−1.61; +2.74 cm) and lower limbs (−1.43; +1.95 cm) [50]. Similarly, Busic showed significant differences between means in 9 out of 15 circumferences (height, waist, hip, chest, upper arms, forearms, and right upper leg circumferences) with a *p* < 0.05, with small differences except for the breast and chest girths, probably due to the chest movements during breathing [49]. Unlike other authors, Lu used the mean absolute difference (MAD) between the DA and CA as a measure of accuracy in addition to the paired *t*-test, finding significant differences in 8 of the 12 body dimensions, *p* < 0.05 [51]. Busic showed good agreement and no significant biases between DA and CA, as analysed by Bland–Altman plots [49]. Kennedy et al. found significant mean differences between three DA devices and CA circumferences on a sample of young children (Δ mean: Fit3D Proscanner^®^ (Redwood City, CA, USA), 1.2–4.2 cm; Styku S100 scanner^®^ (Los Angeles, CA, USA), 1.0–5.5 cm; Size Stream SS20 (Cary, NC, USA), 1.6–3.4 cm; *p* < 0.01). The Fit3D Proscanner^®^ (Redwood City, CA, USA) generally overestimated waist, right arm, and left arm measurements by ~1.5 cm and hip measurements by about 4.0 cm; in contrast, thigh circumferences > 40 cm were generally underestimated. The Size Stream SS20 (Cary, NC, USA) also showed a slight positive bias for waist, hip, right arm, and left arm measurements ranging from 1.6 to 2.5 cm, whereas thigh circumferences were underestimated by ~3.0 cm. Finally, the Styku S100 scanner® (Styku, Los Angeles, CA, USA) prediction bias was less homogenous between different measurement locations [42].

Bourgeois evaluated four circumferences (waist, hip, right arm, and right thigh circumferences) with one time-of-flight scanner (Styku S100 scanner® (Styku, Los Angeles, CA, USA)) and two structured light scanners (KX-16 scanner^®^ (TC LABS, Apex, NC, USA) and Fit3D Proscanner^®^ (Redwood City, CA, USA)) and found differences between the means of the reference methods and DA depending on the scanner used. There were significant differences with KX-16 scanner^®^ (TC LABS, Apex, NC, USA) for hip, right arm, and right thigh circumferences (*p* < 0.0001); with Fit3D Proscanner^®^ (Redwood City, CA, USA) for waist, right thigh (*p* < 0.0001) and hip (*p* < 0.01) circumferences; and with Styku S100 scanner® (Styku, Los Angeles, CA, USA) for waist and right arm circumferences (*p* < 0.0001). In the Bland–Altman analysis, significant biases were found for waist circumference with KX-16 scanner^®^ (TC LABS, Apex, NC, USA) (R^2^ 0.04, *p* < 0.05) and Styku S100 scanner® (Styku, Los Angeles, CA, USA) (R^2^ 0.12, *p* < 0.01); for hip circumference with Fit3D Proscanner^®^ (Redwood City, CA, USA) (R^2^ 0.11, *p* < 0.01); for right arm with KX-16 scanner^®^ (TC LABS, Apex, NC, USA) (R^2^ 0.05, *p* < 0.05) and Styku S100 scanner^®^ (Los Angeles, CA, USA) (R^2^ 0.17, *p* < 0.0001); and for right thigh with KX-16 scanner^®^ (TC LABS, Apex, NC, USA) (R^2^ 0.27, *p* < 0.0001), Fit3D Proscanner^®^ (Redwood City, CA, USA) (R^2^ 0.06, *p* < 0.05) and Styku S100 scanner^®^ (Los Angeles, CA, USA) (R^2^ 0.24, *p* < 0.0001) [34].

Pepper et al. found no significant differences for waist, hip, or waist: hip ratio according to paired-samples *t* tests (*p* = 0.05) [31]. Garlie also found no significant differences between the means of height, weight, neck circumference, and waist circumference [26].

In terms of body shape, DA and CA agreed on popular indices of body shape (waist circumference, waist to hip ratio, waist to height ratio). The correlation was very high [24,25], but the Bland–Altman plot exhibited a bias and a trend towards values in the upper part of the range in DA [25].

### 3.3. Comparison between Digital Anthropometry (DA; 3D Scanners) and Classic Manual Anthropometry (CA) Measurements: Body Composition, Volume, FM and FFM

Numerous studies compared the reliability and accuracy (validity) of body composition estimated using DA vs. specific reference methods (Dual-Energy X-ray Absorptiometry, DXA, Air Displacement Plethysmography, ADP, Bioelectrical Impedance Analysis, BIA, Hydrostatic Weighing, HW) [26,30,32,33,34,35,36,37,39,40,41,44,45,46,47].

As for anthropometric measurements, the results are analysed separately on the basis of the reliability and accuracy criteria, which quantify random and systematic (bias) errors related to anthropometric measurements, respectively (Box 1 and Box 2). The results of studies included in this systematic review are summarized in Table 2, Table 4, Table 5 and Table 6; the comparisons between studies are described in Supplementary Materials Tables S6 and S7.

#### 3.3.1. Reliability

Few studies have reported %TEM (RMS-% CV) reliability. Tinsley Adamson et al. demonstrated that Naked 3D Fitness Trackers^®^ (Redwood City, CA, USA), Styku S100 scanner® (Styku, Los Angeles, CA, USA), and Size Stream SS20^®^ (Cary, NC, USA) were the most accurate devices for estimating body fat percentage (%BF) (RMS-% CV 2.3–2.9%), with FIT3D Proscanner^®^ (Redwood City, CA, USA) showing slightly higher errors (RMS-% CV: 4.0–4.3%). Similar precision was reported for FM (kg), while FFM exhibited an RMS-%CV of 0.7–1.4% for all scanners [41]. Another study by Tinsley et al. using the same scanners reported an average RMS-% CV of 1.9–2.3% for body volumes; the lowest value was observed for total body volume (RMS-% CV < 1% for all scanners), followed by trunk (~1.2%), legs (~2.5%), and arms (~3 to 5%) [47].

Relative reliability indices showed concordant results: Tinsley Adamson et al. reported high ICCs 0.975–0.999 (*p* < 0.0001) for %BF, FM, and FFM [41]; the same group reported comparable reliability with reference to body volumes (ICCs 0.952–0.999 for body volumes) [47]. Pepper et al. observed ICCs ≥ 0.99, with thigh volume being the most reliable (ICC = 0.997) [32], and Wang confirmed ICCs > 0.97 for body volumes [46].

In terms of absolute reliability, Tinsley Adamson reported that Naked 3D Fitness Trackers^®^ (Redwood City, CA, USA), Styku S100 scanner® (Styku, Los Angeles, CA, USA), and Size Stream SS20^®^ (Cary, NC, USA) were highly reliable for %BF (Precision Error = SEM = 0.5–0.7%), with FIT3D Proscanner^®^ (Redwood City, CA, USA) showing slightly higher errors (PE = SEM = 1.0–1.1%). Similar results were reported for FM (kg) [41]. With a time-of-flight (ToF) scanner, for %BF, Harbin found SEM = 0.469 between DA and BIA and SEM = 0.307 between DA and HW [33]. Ng et al. observed %CV < 5% for volumes (0.91–4.49%) but a higher CV% in derived regional FM and FFM (Visceral Adipose Tissue (VAT): 6.69%; Arms FFM: 6.67%; Arms FM: 11.63%) [45]. Wong reported precise 3D scan measurements, particularly for fat mass (CV 3.30%) and fat-free mass (CV 1.34%) [37]. Bourgeois made a comparison among 3D, DXA, and ADP precision, and the CV values of reference methods (DXA and ADP) did not exceed 1.5%, indicating good reliability; in regional body volumes, 3D scans showed higher CVs (0.3–5.7%), which were less with Styku S100 scanner® (Styku, Los Angeles, CA, USA) than with the reference method for the trunk and left leg [34]. According to Pepper et al., the CVs were all <5%, with total body volumes (BV) being the most reliable (CV 0.41%) and thigh volume being the most variable (CV 2.26%) [32]. Similar results were observed by Kennedy et al. with %BF consistent among repeated measurements (CV = 2.4%) [39]. Ng et al. found a higher CV% in derived regional FM and FFM (VAT: 6.69%; Arms FFM: 6.67%; Arms FM: 11.63%) than in volumes (0.91–4.49%) [45].

#### 3.3.2. Accuracy

Several studies examined the relationships between total or segmental BV and body composition using 3D body scanners with those by BIA, DXA, ADP, and HW [26,30,32,33,34,35,36,37,39,41,43,44,45,46,47].

The measurement results are presented below as the minimum and maximum values; the range can be wide depending on the specific body segments and the laser used to measure them.

For %BF, good correlation was reported for DA and other body composition methods: DXA (r ≥ 0.74) [26,36], HW (r = 0.816) [33], ADP (r = 0.899) [35] and BIA (r = 0.888) [33]. Garlie evaluated the correlation through CCC, and the values were moderate and statistically significant (CCC = 0.74, *p* < 0.05) [26].

Cabre and Milanese examined body composition. In Cabre’s study there was a strong correlation between 3D, DXA, and four compartments (4C) model for FM (3D vs. DXA r = 0.90, 3D vs. 4C r = 0.85) and for FFM (3D vs. DXA r = 0.90, 3D vs. 4C r = 0.92) [36]. Milanese et al. compared post-exercise changes in total and regional FM, as detected by DA and DXA. Whole-body FM showed a fair linear correlation with DXA counterparts (r > 0.5); 4 out of 6 regional DA trunk FM changes correlated with DXA measurements. As for relative changes, only total %FM and trunk %FM correlated with their respective DA measurements [30].

The association between DA and other methods was studied through the coefficient of determination. For total body volume, a strong correlation between DA, ADP, and DXA (R^2^ > 0.97, RMSE 1.618–9.7) was described [34,40,45].

Despite a strong prediction of a total BV R^2^ = 0.98–1.0, Tinsley et al. observed significant overestimation (Size Stream SS20^®^ (Cary, NC, USA)) and underestimation (Styku S100 scanner® (Styku, Los Angeles, CA, USA)) (both *p* < 0.01) for the DA vs. the reference method (4C model). The reported RMSE for total BV ranged from 4.2 to 10.5 liters depending on the scanner, whereas DXA predictive equations showed a RMSE of 0.7–1.5 L. Compared with the 4C model, Bland–Altman plots showed systematic proportional bias in total BV (with statistically significant regression coefficients) for all four scanners and wider LoA with the 4C model than with DXA (LoA 2.9–5.3 L vs. 1.1–2.0 L, DA vs. DXA). Similar accuracy issues were also reported for regional volumes, with DA significantly overestimating trunk volume as well as underestimating both arm and leg volumes. Furthermore, all 3D regional volumes failed to exhibit equivalence with DXA-derived volumes [47].

Total-body volumes measured by the three-dimensional photonic scanner (3DPS) and underwater weighing (UWW) were linearly correlated (R^2^ = 0.999 and SEE = 0.892 L), with DA showing significantly greater values (*p* < 0.001) [46].

Bourgeois reported all significant correlations with DXA for regional body volume measured by DA (R^2^ 0.69–0.98, *p* < 0.0001, RMSE 0.8–14.0) [34]. DXA regional volume estimates showed moderate prediction by DA (R^2^ = 0.73–0.97), with the latter including less volume in the limbs and relatively more volume in the trunk compartment (all *p* < 0.001) [45]. Sobhyeh reported a good correlation between DA and DXA (arms: R^2^ 0.75 vs. 0.79, legs: R^2^ 0.86 vs. 0.89, trunk: R^2^ 0.97 vs. 0. 98, for Styku S100 scanner® (Styku, Los Angeles, CA, USA) vs. SS20 (Cary, NC, USA)) [40]; similarly, Wong reported values of R^2^ > 0.7 (arm: R^2^ 0.962, RMSE 0.255; leg: R^2^ 0.763, RMSE 2.159; trunk: R^2^ 0.968, RMSE 1.683) [37].

Lower values between 3D and DXA for %BF were reported by Kennedy (R^2^ 0.73, *p* < 0.0001) [39] and by Cabre (R^2^ 0.74, SEE 4.20) [36]. Cabre also reported on correlations between 3D and DXA for FM (R2 0.81, SEE 2.91) and FFM (R2 0.88, SEE 3.77) and between the 3D and 4C model for %BF (R2 0.63, SEE 5.31), FM (R2 0.72, SEE 3.64), and FFM (R2 0.84, SEE 4.76) [36]. Garlie reported SEE 3.2 for %BF between 3D and DXA [26]; Wagner found a significant (*p* < 0.001) overestimation of %BF by DA compared with ADP with R^2^ = 0.809 and SEE 4.13% [35], and Wong reported a strong association between 3D and DXA for %BF (R^2^ 0.855, RMSE 3.63) [37].

Using (UWW as the criterion method, Wang et al. found no significant difference in %BF between DA and UWW (*p* = 0.4801), although the absolute differences were higher than in volumes [46].

In a study by Tinsley et al., three body scanners employed (Size Stream SS20^®^ (Cary, NC, USA), FIT3D Proscanner^®^ (Redwood City, CA, USA), and Naked 3D Fitness Trackers^®^ (Redwood City, CA, USA)) were within the 5% equivalence region of a 4C model in terms of %FM, FM, and FFM (±1.3% body fat, ±1.0 kg FM, and ±2.7 kg FFM). The CCCs of all the scanners were 0.74–0.90 (%FM), 0.85–0.95 (FM), and 0.93–0.97 (FFM). FIT3D Proscanner^®^ (Redwood City, CA, USA) displayed the lowest RMSE for all variables (2.8 kg for FM and FFM; 3.7% for %FM), and Naked 3D Fitness Trackers^®^ (Redwood City, CA, USA) and Size Stream SS20^®^ (Cary, NC, USA) displayed slightly higher values (~3.7 kg for FM and FFM; 4.8% for %FM); Styku S100 scanner® (Styku, Los Angeles, CA, USA) displayed the largest RMSE (4.6 kg for FM and FFM; 6.1% for %FM). According to the Bland–Altman analysis, FIT3D Proscanner^®^ (Redwood City, CA, USA) showed the narrowest LoA (±7% for %BF and ±~5.5 kg for FM and FFM), with other scanners showing larger values (±9.0–9.5% for %BF and ±~7.0 kg for FM and FFM). Proportional bias was largest for %FM, with regression coefficients ranging from ±0.1 to 0.3 for all scanners (all *p* < 0.01). On the one hand, only Naked 3D Fitness Trackers^®^ (Redwood City, CA, USA) did not display proportional bias for FM, while the other scanners displayed regression coefficients of 0.1 to 0.2 (*p* < 0.0001). On the other hand, only Styku S100 scanner® (Styku, Los Angeles, CA, USA) did not display proportional bias for FFM, while all other scanners exhibited proportional bias, which was statistically significant but small (coefficients ± 0.1) [41].

In a study by Sobhiyeh, total FM by DA calculated with Siri’s equation strongly agreed with DXA (R^2^ = 0.84 vs. 0.86, Styku S100 scanner® (Styku, Los Angeles, CA, USA) vs. Size Stream SS20 (Cary, NC, USA)). Appendicular FM estimated by the universal software also agreed with DXA (R^2^ 0.72–0.88 for Styku S100 scanner® (Styku, Los Angeles, CA, USA) and R^2^ 0.76–0.85 for Size Stream SS20 (Cary, NC, USA)), although less well than with ADP values [40].

In another study using DXA as a reference method for %BF, android and gynoid FM showed higher prediction values for android and gynoid FM (R^2^ 93.2% android and 91.4% gynoid) than %BF (76.4% android and 66.5% gynoid). As for Bland–Altman plots, both FM and %FM data were randomly dispersed within the 95% LoA; the limits of agreement for FM and %FM were −0.06 ± 0.87 kg and −0.11 ± 1.97 % for android and −0.04 ± 1.58 kg and −0.19 ± 4.27% for gynoid, respectively; few outliers and a systematic bias ~0 cm for both android and gynoid FM were observed [44].

The mean differences between DA and other methods were measured. When measuring total body volume with DA, Bourgeois reported a significant (*p* < 0.0001) underestimation in comparison with DXA and ADP; for regional body volume, Bourgeois found significant differences: the volume of the trunk was overestimated, and the volumes of arms and legs were underestimated [34].

The 3D estimates of %BF, FM and FFM were significantly (*p* < 0.001) different compared with those from 4C (3D-4C: %BF −4.13, FM −2.66, FFM 3.15), but not significantly different compared with those from DXA (3D-DXA: %BF 0.1, FM 0.28, FFM 0.10). With high mean differences and wide limits of agreement (%BF: −6.39–14.64; FM −4.47–9.79; FFM −14.9–3.78), the Bland–Altman plot confirmed the overestimation of %BF measured with 3D compared with 4C, especially in subjects with increased adiposity; however, when compared with DXA, the estimates were more acceptable, even if the LoA were quite wide (%BF: −8.46–8.25; FM: −5.99–5.42; FF: −7.68–7.98) [36]. The results appeared similar to a study by Harbin, who examined %BF and revealed a proportional bias in DA comparing circumferences (LoA: −12.22 to 5.822), BIA (LoA: −10.12 to 6.213) and HW (LoA: −14.51 to 5.104) and reduced accuracy among subjects with increased adiposity [33]. Similarly, Wagner demonstrated significant bias (r = −0.597, *p* < 0.001) in the estimates of %BF in the Bland–Altman plot, overestimation of thinner participants and underestimation of fatter participants, with the LoA between −6.7 and 11% [35].

Garlie showed small and no significant mean differences between DA and DXA of 0.11 ± 3.1%, with a LoA ranging from −6.06 to 6.28% [26]. Pepper reported no significant difference between DA and DXA, and HW measured %BF [32]. In the study by Kennedy et al., the Naked Body Scanners (Naked Labs Inc, Redwood City, CA, USA) showed a trending bias to underestimate %FM in individuals with less than ~30% body fat (*p* = 0.09) [39].

Similarly, Wong reported a good agreement between DA and DXA for %BF and a proportional bias for regional body volumes with an overestimation for total volume and volumes of arm and leg, and an underestimation for trunk volume [37]. In contrast, Bourgeois reported a proportional bias and an overestimation for trunk volume with three scanners compared with DXA (KX-16 scanner^®^ (TC LABS, Apex, NC, USA): R^2^ 0.31; Fit3D Proscanner^®^ (Redwood City, CA, USA): R^2^ 0.78; Styku S100 scanner® (Styku, Los Angeles, CA, USA): R^2^ 0.32) and an underestimation for total body volume compared with ADP (KX-16 scanner^®^ (TC LABS, Apex, NC, USA)): R^2^ 0.31; Fit3D Proscanner^®^ (Redwood City, CA, USA): R^2^ 0.18; Styku S100 scanner® (Styku, Los Angeles, CA, USA): R^2^ 0.69) [34].

### 3.4. Certainty in the Evidence

The quality of evidence was assessed by means of the GRADE tool [23]. The GRADE approach rates each outcome across studies, assigning a final grade of “high”, “moderate”, “low”, or “very low” for all critically important outcomes. Clinical parameters of anthropometric measures and body composition were used. The certainty of evidence was considered very low for almost all studies: nineteen studies [24,25,26,28,29,30,33,35,37,38,39,40,43,44,47,48,50,51] had a serious risk of bias according to the AXIS tool, three studies [27,42,45] were considered imprecise for the narrow sample size, and two studies [36,42] used an indirect comparison of evidence. Four studies were rated low [24,26,29,48] (Table 7).

## 4. Discussion

This work focused on how three-dimensional body scanners perform in terms of anthropometric measurements and body composition estimates. The majority of included studies reported good reliability and accuracy of DA, with laser-based scanners outperforming other technologies [24]. SL-projectors and ToF scanners produced a wide spectrum of results: some studies found lower, though still acceptable, reliability than reference methods [34,37,38,48], whereas two studies reported poor precision in both CA and SL-projector or ToF scanners [39,49], and one study demonstrated better precision with SL-projector and passive stereo scanners than the reference method [34].

With the exception of one [26], all the studies agreed on a good correlation between traditional and 3D body composition estimates, but 3D imaging showed a systematic bias. In particular, because of heterogeneity in landmark positioning and body surface partitioning algorithms [33], three of the studies included in the review found less accurate estimation of the %BF and total BV by 3D imaging than BIA, HW, and ADP among adults with increased adiposity [33,34,35].

Despite this observation, DA appears to be less time-consuming and more reliable than CA, especially in the clinical population with obesity. Furthermore, three-dimensional body scanners have some significant advantages: they are more affordable than DXA, which requires adequately trained personnel in the acquisition and post-processing phases, and exposes patients to ionizing radiation. They are also less expensive and invasive than other reference body composition techniques (ADP, HW) in the field of bicompartmental models (Fat Mass and Fat Free Mass) of human body composition assessment.

However, DA has a few limitations due to technical and human variability. Technical variability is influenced by the characteristics of 3D scanning hardware and the performance of data acquisition, visualization, landmarking, and measurement extraction software. Stationary SL laser scanners show sub-millimeter accuracy and resolution, although their cost and slow scanning time limit their use to experimental settings. At the other end of the spectrum, passive stereo (PS) handheld devices or ToF mini-scanners represent economical “field measurement” options, though lower resolution limits their use in collecting ground-truth data [53].

Currently, the absence of validated reference software makes the use of patent-protected technologies and software from different manufacturers, which only allow updates and calibrations but no direct comparison between different models, unavoidable [54]. As a first step towards DA standardization, several studies have proposed the development of standard software (which does not require laborious manual positioning of reference landmarks), paving the way for cross-validation of body measurements across different devices [40,43]. Furthermore, incorporating principal component analysis (PCA) into regression models trained by machine-learning algorithms could lead not only to improved accuracy of body composition estimates but also of haematological metabolic parameters, muscle strength, and performance [55,56]. Finally, from a global rehabilitation perspective, the integration of postural analysis based on 3D imaging in a complete tool for assessing patients’ nutritional status could provide useful diagnostic information to researchers or clinicians, considering that patients with over- or under-nutrition (such as obesity and/or eating disorders) may be affected by pathologies affecting the musculoskeletal system [57,58,59,60].

Participants ability to minimize motion artifacts and to replicate a standard pose across several scans is contributes to human variability [54]. Indeed, experimental studies have demonstrated better accuracy and precision if the human variation is under the control of the experimenters. Lu et al. used a dummy to eliminate interference from body sway and allow for stable posture. They showed that the mean values of the absolute difference between the scan-derived measurements and hand-held measurements, and between the scan-derived repeated measurements were better than the mean values reported in other studies that did not use a dummy [51]. It is, therefore, recommended to normalize the rate and depth of respiration during the acquisition phase through repeated measurements; otherwise, serious accuracy problems may arise. To minimize the impact of posture on human variability, without compromising the quality of the scans, positioning aids have been developed [54].

Furthermore, if a standardized pose is not adopted by the subjects analysed, it may be possible to remove the unwanted pose variance (i.e., a random error introduced by different postures) by rigging individual 3D meshes to a standard pose. This in turn would improve the mathematical models applied to the prediction of human body composition [37,56].

Additional clinical applications of 3D body scanners include anorexia nervosa and obesity diagnosis and treatment. The ability of patients to describe other people’s 3D body images and their own body images could help clarify any relationship between the mental representation of the body and body image distortion [61].

### Limitations

Our systematic review suffers from a number of drawbacks. The included studies are observational (cross-sectional), lowering the overall quality of evidence compared with experimental studies. The study sample sizes were generally small, with heterogeneous ages, ethnic groups, and body mass index (BMI) classes. The heterogeneity of measurement sites further limits the comparability of studies. As mentioned above, digital scanners used patent-protected technologies and software from different manufacturers, which limits direct comparisons between devices; finally, the “reference” methods used in DA validation were not always gold standard techniques.

## 5. Conclusions

Initially designed for the textile industry, DA applications have now expanded to human nutrition, due to rapid technological advancements. The high reliability and speed of measurement detection make DA more suitable than its conventional counterparts in specific contexts, e.g., large-scale population surveys or clinical subpopulations. Furthermore, 3D body imaging could be used in place of other known methods of body composition assessment where biological costs (DXA, computed tomography (CT)) or technical/time constraints (ADP, UWW, magnetic resonance (MR)) are of concern. Finally, AD could be proposed as a screening tool before second-level imaging techniques for the assessment of human body composition as well as in postural analysis. However, hardware variability, a lack of standard validated software, the cost of more accurate and precise scanners, and small sample sizes limit the quality of the evidence in current studies.

For all of these reasons, this systematic review of the literature was able to achieve the primary objective of providing an update on the state of digital anthropometry. The secondary objective was to verify the methods for identifying the best technology to be used in the field of DA, to identify how technologies can be selected appropriately for specific applications, and to identify ways in which digital anthropometry technologies can be incorporated into daily clinical practice. Investigation of this objective highlights a series of concerns that must first be further investigated in order to address the above.

Finally, although the contribution of anthropometric measurements in statistical models for the prediction of human body composition (for example, in the estimation of lean body mass) is extremely high and explains over 80% of variability [62], to explain residual variability, especially in different clinical settings, it is necessary to develop new tools and software that integrate the available analytical methods of human body composition according with the perspective of multicompartmental models.

## Figures and Tables

**Table 1:** Characteristics of the included studies.

Authors and Year of Publication	Study Design	Country	Number of Participants	Average Age (Year ± SD)			Males	Males %	Average Age (Year ± SD)			Females	Females %	Average Age (Year ± SD)		
Harbin et al. (2018) [33]	Cross-sectional	USA	265	22.1	±	2.5	119	45%	22.4	±	2.7	146	55%	21.8	±	2.4
Bourgeois et al. (2017) [34]	Cross-sectional	USA	113	44	±	17	40	35%	41	±	17	73	65%	47	±	17
Koepke et al. (2017) [24]	Cross-sectional	Switzerland	123	24.55	±	4.18	123	100%				0	0%			
Garlie et al. (2010) [26]	Cross-sectional	USA	37	28.4	±	12.7	37	100%				0	0%			
Wagner et al. (2019) [35]	Cross-sectional	USA	79	32.9	±	12.4	42	53%	33.2	±	11.9	37	47%	32.5	±	13.1
Busic et al. (2020) [49]	Cross-sectional	Slovenia	51	NA			12	24%	NA			39	76%	NA		
Sager et al. (2020) [25]	Cross-sectional	Switzerland	104	20.5	±	1.1	104	100%				0	0%			
Japar et al. (2017) [28]	Cross-sectional	Malaysia	200	29.83			0	0%				200	100%	NA		
Cabre et al. (2021) [36]	Cross-sectional	USA	194	23.52	±	5.47	83	43%	23.8	±	6.1	111	57%	23.2	±	4.9
Wells et al. (2015) [48]	Cross-sectional	UK	1022	8.44	±	1.57	NA					NA				
Wong et al. (2019) [37]	Cross-sectional	USA	112	12.5	±	3.3	46	41%	12.3	±	3.3	66	59%	12.7	±	3.3
Conkle et al. (2018) [38]	Cross-sectional	USA	474	2.17			246	52%	NA			228	48%	NA		
Heuberger et al. (2008) [29]	Cross-sectional	USA	85	19.5	±	1.4	0	0%				85	100%			
Lu et al. (2010) [51]	Cross-sectional	China	263	18–30			172	65%	NA			91	35%	NA		
Beckmann et al. (2019) [27]	Cross-sectional	Switzerland	52	19–23			52	100%	NA			0	0%			
Kennedy et al. (2020) [39]	Cross-sectional	USA	90	7–74			36	40%	NA			54	60%	NA		
Sobhiyeh, Dunkel et al. (2021) [40]	Cross-sectional	USA	356	21–79			155	44%	NA			201	56%	NA		
Tinsley, Benavides et al. (2020) [47]	Cross-sectional	USA	179	33.6	±	15.3	76	42%	33.8	±	14.5	103	58%	33.4	±	15.9
Kennedy et al. (2022) [42]	Cross-sectional	USA	64	5–8			29	45%	6.9	±	1.1	35	55%	6.6	±	1.2
Tinsley, Adamson et al. (2020) [41]	Cross-sectional	USA	171	33.1	±	15.2	75	44%	NA			96	56%	NA		
			139				65	47%	NA			74	53%	NA		
Milanese et al. (2015) [30]	Cross-sectional	Italy	25	20–60			0	0%				25	100%			
Pepper et al. (2010) [31]	Cross-sectional	USA	70	30.91	±	1.31	0	0%				70	100%			
Pepper et al. (2011) [32]	Cross-sectional	USA	70	29.74	±	1.41	0	0%				70	100%			
Sobhiyeh, Kennedy et al. (2021) [43]	Cross-sectional	USA	35	NA			23	66%	22.7	±	2.9	12	34%	22	±	3.2
Lee et al. (2015) [44]	Cross-sectional	USA	121	34.38	±	0.98	67	55%	NA			54	45%	NA		
Ng et al. (2016) [45]	Cross-sectional	USA	39	44.3	±	15.5	20	51%	NA			19	49%	NA		
			37				18	49%	NA			19	51%	NA		
Simenko et al. (2016) [50]	Cross-sectional	Slovenia	31	22.1	±	4.63	17	55%	NA			14	45%	NA		
Wang et al. (2006) [46]	Cross-sectional	USA	92	6–83			44	48%	NA			48	52%	NA		

Abbreviations: SD = standard deviation; NA = not available.

**Table 2:** Summary table of included studies.

Authors and Year of Publication	Scanner Utilised	DA Technology	Comparison Method
Harbin et al. (2018) [33]	Styku body scanner (MYBODEE^TM^, Styku, Los Angeles, CA, USA)	time-of-flight	Manual measurements, BIA and hydrostatic weighing
Bourgeois et al. (2017) [34]	KX-16 scanner (TC LABS, Apex, NC, USA) Styku S100 scanner (Styku, Los Angeles, CA, USA) Fit3D Scanner (Redwood City, CA, USA)	structured light time-of-flight structured light ir	Manual measurements, DXA, ADP
Koepke et al. (2017) [24]	BS Vitus Smart XXL (Human solution GmbH, Kaiserslautern, Germany)	structured light laser	Manual measurements
Garlie et al. (2010) [26]	Cyberware WB4 laser body scanner (Cyberware Inc., Monterey, CA, USA)	structured light laser	Manual measurements and DXA
Wagner et al. (2019) [35]	Fit3D Scanner (Redwood City, CA, USA)	structured light ir	Manual measurements and ADP
Busic et al. (2020) [49]	BodyRecog mobile 3D scanner (BodyRecog Metrics, Boston, MA, USA)	structured light ir	Manual measurements
Sager et al. (2020) [25]	Anthroscan VITUS body scan (Human solution GmbH, Kaiserslautern, German)	structured light laser	Manual measurements
Japar et al. (2017) [28]	NX-16 body scanner (Cary, NC, USA)	structured light white light	Manual measurements
Cabre et al. (2021) [36]	Styku body scanner (MYBODEE^TM^, Styku, Los Angeles, CA, USA)	time-of-flight	BIA, DXA, ADP
Wells et al. (2015) [48]	NX-16 body scanner (Cary, NC, USA)	structured light white light	Manual measurements
Wong et al. (2019) [37]	Fit3D Proscanner^®^ (Redwood City, CA, USA)	structured light ir	Manual measurements and DXA
Conkle et al. (2018) [38]	AutoAnthro scanner (Occipital, San Francisco, CA, USA)	structured light white light	Manual measurements
Heuberger et al. (2008) [29]	VITUS Smart 3D (Human solution GmbH, Kaiserslautern, Germany)	structured light laser	Manual measurements
Lu et al. (2010) [51]	Vitronic Vitus-3D 1600 scanning system (Human solution GmbH, Kaiserslautern, Germany)	structured light laser	Manual measurements
Beckmann et al. (2019) [27]	VITUS body scan (Human solution GmbH, Kaiserslautern, Germany)	structured light laser	Manual measurements
Kennedy et al. (2020) [39]	Naked Body Scanner (Naked Labs Inc., Redwood City, CA, USA)	structured light ir	Manual measurements, DXA
Sobhiyeh, Dunkel et al. (2021) [40]	Styku S100 (Los Angeles, CA, USA) Size Stream SS20 (Cary, NC, USA)	time-of-flight structured light ir	ADP and DXA
Tinsley, Benavides et al. (2020) [47]	Naked Labs 3D Fitness Tracker (Redwood City, CA, USA) Fit3D Proscanner^®^ (Redwood City, CA, USA) Size Stream SS20 (Cary, NC, USA) Styku S100 (Styku, Los Angeles, CA, California)	structured light ir structured light ir structured light ir time-of-flight	ADP and DXA
Kennedy et al. (2022) [42]	Fit3D Proscanner^®^ (Redwood City, CA, USA) Size Stream SS20 (Cary, NC, USA) Styku S100 (Styku, Los Angeles, CA, USA)	structured light ir structured light ir time-of-flight	Manual measurements
Tinsley, Adamson et al. (2020) [41]	Naked Labs 3D Fitness Tracker (Redwood City, CA, USA) Fit3D Proscanner^®^ (Redwood City, CA, USA) Size Stream SS20 (Cary, NC, USA) Styku S100 (Styku, Los Angeles, CA, USA)	structured light ir structured light ir structured light ir time-of-flight	4-component (4C) model (BIA-BIS, DXA, ADP, scale)
Milanese et al. (2015) [30]	Breuckmann GmbH Body-SCAN (Breuckmann GmbH, Meersburg, Germany)	structured light white light	DXA
Pepper et al. (2010) [31]	Rotatory Laser Body scanner (by Bugao Xu) (University of Texas, Austin, TX, USA)	structured light laser	DXA, hydrostatic weighing
Pepper et al. (2011) [32]	Rotatory Laser Body scanner (by Bugao Xu) (University of Texas, Austin, TX, USA)	structured light laser	Manual measurements
Sobhiyeh, Kennedy et al. (2021) [43]	Fit3D Proscanner^®^ (Redwood City, CA, USA) Size Stream SS20 (Cary, NC, USA) Styku S100 (Styku, Los Angeles, CA, USA)	structured light ir structured light ir time-of-flight	Manual measurements
Lee et al. (2015) [44]	Stereovision body imaging (prototype by Bugao Xu) (University of Texas, Austin, TX, USA)	passive stereo	Manual measurements
Ng et al. (2016) [45]	Fit3D Proscanner^®^ (Redwood City, CA, USA)	structured light ir	Manual measurements
Simenko et al. (2016) [50]	NX-16 body scanner (Cary, NC, USA)	structured light white light	Manual measurements
Wang et al. (2006) [46]	C9036-02 (Hamamatsu Photonics KK, Hamamatsu, Japan)	structured light laser	Manual measurements, hydrostatic weighing

Abbreviations: DA = digital anthropometry; ir = infrared; BIA = bioimpedance analysis; DXA = dual-energy X-ray absorptiometry; ADP = air displacement plethysmography; BIS = bioimpedance spectroscopy.

**Table 3:** Statistical analysis of included studies evaluating classic anthropometric measurements: circumferences, lengths and shape.

Authors and Year of Publication	Summary Statistics	Results		Conclusion
		Reliability	Accuracy	
Koepke et al. (2017) [24]	Reliability: Technical Error of Measurement (TEM), intraclass correlation coefficient (ICC) Accuracy: Pearson’s coefficient (r), concordance correlation coefficient (CCC), mean difference (groups), *t*-test, Bland–Altmann plot	No significant differences between repeated DA and repeated CA, except for CA for chest and waist. TEM in DA (height: 0.45, chest: 1.24, waist: 0.98, buttock 1.18, hip: 1.05) than in CA (height: 0.50, chest: 8.19, waist: 4.36, buttock: 6.84, hip: 2.50); ICCs in CA (chest: 0.968, waist: 0.990, buttock: 0.955, hip: 0.972) and DA (chest: 0.981, waist: 0.993, buttock: 0.997, hip: 0.994), for height (in CA 0.999, in DA 0.998).	Correlation between methods (r between 0.933 and 0.993, CCC between 0.718 and 0.960); buttock circumference (r = 0.828, CCC = 0.258) and significant mean differences (height: −2.01, *p* < 0.001; chest: 3.88, *p* < 0.001; waist: 1.17, *p* < 0.001; buttock: 12.62, *p* < 0.001; hip: 4.37, *p* < 0.001); Body shape: significant difference between two methods (WHR: −0.03, *p* < 0.001; WHtR: 0.01, *p* < 0.001; BMI: 0.52, *p* < 0.001); correlation (r 0.979–0.996, CCC 0.920–0.974); WHR (r = 0.857, CCC = 0.673).	The precision and the intraclass correlation coefficients were better in DA than in CA, and the two methods were highly correlated, but there were significant differences between two methods.
Busic et al. (2020) [49]	Reliability: coefficient of variation (CV), standard error of estimate (SEM) Accuracy: Pearson’s coefficient (r), coefficient of determination (R^2^), mean difference (groups), *t*-test, Bland–Altman plot	Analysis of 15 measures, CVs were >5%, except for the hip circumference (in DA = 4.243%; in CA = 4.295%), and higher in DA (5.265%–10.291%) than in CA (5.090%–10.178%). SEM values were similar between two methods, higher in chest and breast circumferences.	The correlation measured by r (0.865–0.995) and R^2^ (0.749–0.990) was high in almost all measurements. Significant differences between means in 7 measurements (breast, right and left wrist, right and left upper leg, right and left lower leg circumference) of 15 (*p* < 0.005). Bland–Altman plots indicated good agreement.	CV values do not demonstrate good performance. The agreement between the methods was good, but there were significant differences in over half of the measurements.
Sager et al. (2020) [25]	Accuracy: Spearman Rho, mean difference (groups), Bland–Altman plot		Strong correlation between DA and CA for the height (Spearman Rho = 0.98), waist circumference and WHtR (Spearman Rho = 0.96) and BMI (Spearman Rho = 1). Bland–Altman plot indicated a constant bias for the height and a trend in the upper part of the range in DA than CA.	Body measurements obtained with both methods were strongly correlated; there was a constant bias for DA measures
Japar et al. (2017) [28]	Accuracy: mean difference (groups), *t*-test		Significant differences between DA and CA for waist (in DA: 85.34 ± 13.36, in CA: 84.63 ± 13.82, *p* < 0.05) and hip circumferences (in DA: 103.47 ± 11.53, in CA: 94.88 ± 15.08, *p* < 0.01) and waist to hip ratio (in DA: 0.82 ± 0.02, in CA: 0.89 ± 0.04, *p* < 0.01).	The DA produced higher readings in waist and hip circumferences compared with CA.
Wells et al. (2015) [48]	Reliability: technical error of measurement (TEM) Accuracy: Pearson’s coefficient (r), coefficient of determination (R^2^), standard error of estimate (SEE), mean difference (groups), *t*-test, Bland–Altmann plot	TEM higher in DA (circumference of chest: 1.57, waist: 1.49, knee: 1.36, calf: 0.90) than in CA (circumference of chest: 0.13, waist: 0.06, knee: 0.06, calf: 0.04)	Strong correlation between two methods (r ≥ 0.95, R^2^ ≥ 0.89, SEE 0.80–2.37). The mean differences of circumferences (chest: 3.67 ± 2.23, waist: 1.36 ± 2.37, knee: 1.39 ± 1.21, calf: 0.62 ± 0.80) and Bland–Altman plots showed a significant (*p* < 0.0001) and proportional bias.	The reliability was better in CA, and the correlation between DA and CA was strong, but there were biases that varied with outcome size (DA produced larger measures than CA).
Conkle et al. (2018) [38]	Reliability: technical error of measurement (TEM) and percentage technical error of measurement (%TEM), coefficient of reliability (R), intraclass correlation coefficient (ICC) Accuracy: mean difference (groups), paired *t*-test, Bland–Altmann plot	In DA, intraobserver TEM and TEM% (stature 0.62 cm–0.8%, HC 0.41 cm–0.9%, MUAC 0.35 cm–2.3%) and interobserver TEM (stature 0.46 cm–0.5%, HC 0.30 cm–0.7%, MUAC 0.25 cm–1.7%) were higher than in CA intraobserver TEM (stature 0.36 cm–0.4%, HC 0.20 cm–0.4%, MUAC 0.20 cm–1.3%) and interobserver TEM (stature 0.37 cm–0.5%, HC 0.26 cm–0.6%, MUAC 0.24 cm–1.6%). The inter-observer TEM was higher than the intra-observer TEM for CA and not for DA. For CA, total TEM was 0.51 cm for stature, 0.33 cm for HC, 0.31 cm for MUAC, compared with 0.77 cm for stature, 0.51 cm for HC, for 0.43 cm for MUAC for DA. The R and ICC were close to 1.00 for repeated measurements for both techniques.	According to Bland–Altman plots, there were significant differences (*p* < 0.001) for height (mean difference 0.59 and LoA −0.1–1.2), for head circumference (mean difference 0.32 and LoA −0.1–0.8), and for arm circumference (mean difference −0.19 and LoA −0.6–0.2).	The measures were reliable with both methods, but the precision was better in the CA. The agreement was good, but there was significant bias with an overestimation of height (+0.6 mm) and head circumference (+0.3 mm) and an underestimation for arm circumference (−0.2 mm).
Heuberger et al. (2008) [29]	Accuracy: coefficient of determination (R^2^)		The linear regression R^2^ of hip (0.63, *p* < 0.05) and waist-to-hip ratio (0.53, *p* < 0.05) were significant. For waist, height and weight, the same results were not found; significant differences (*p* < 0.01) existed between DA and CA for circumferences of hip (DA: 40 ± 4.5 cm; CA: 39 ± 4.7 cm) and waist (DA: 33 ± 4.2 cm; CA: 32 ± 4.2 cm).	The accuracy of measures of hip and waist-to-hip ratio decreased when the measure increased. DA produced an overestimation of waist and hip circumferences.
Lu et al. (2010) [51]	Accuracy: mean difference, *t*-test		Significant differences between DA and CA in eight of the 12 measurements (shoulder breadth, *p* = 0.0047; anterior chest breadth, *p =* 0.0004; cervical to waist length, *p* = 0.0023; chest circumference, *p* = 0.0008; waist circumference, *p* = 0.0090; sleeve length, *p* = 0.0001; front length, *p* = 0.008; back length, *p* = 0.0167).	The accuracy of DA was lower than of CA, probably due to variations caused by human subjects.
Beckmann et al. (2019) [27]	Accuracy: Pearson’s coefficient (r), Spearman Rho, concordance correlation coefficient (CCC), mean difference, *t*-test, Bland–Altmann plot		The correlation between DA and CA for waist (r = 0.979, CCC = 0.964, Rho = 0.964) and height (r = 0.995, CCC = 0.988, Rho = 0.989) was strong. The mean differences between two methods for waist (−1.50 cm) and height (0.77 cm) were significant (*p* < 0.001). The agreement at the Bland–Altman plot was very good, but there was a systematic bias.	The correlation was good. The waist circumference was systematically smaller in DA than in CA, and height was less in CA than DA
Kennedy et al. (2022) [42]	Accuracy: *t*-test, coefficient of determination (R^2^) of multiple regression, Bland–Altman plot, mean differences (between groups)		All three scanners showed significant mean differences (paired *t*-test, *p* < 0.01) with CA (Δ mean: Fit3D Proscanner^®^ (Redwood City, CA, USA), 1.2–4.2 cm; Styku body scanner (Los Angeles, CA, California), 1.0–5.5 cm; Size Stream SS20 (Cary, NC, USA), 1.6–3.4 cm; *p* < 0.01). The only exception was left thigh measurement by the Fit3D Proscanner^®^ (Redwood City, CA, USA) (Δ mean: 0.3 cm). Linear regression analysis: DA significantly predicted manual measurements (R^2^: Fit3D Proscanner^®^ (Redwood City, CA, USA), 0.70–0.96; Styku S100 scanner (Los Angeles, CA, USA), 0.54–0.97; Size Stream SS20 (Cary, NC, USA), 0.68–0.97; *p* < 0.01), with Fit3D Proscanner^®^ (Redwood City, CA, USA) being the best predictor of body size in small children (R^2^ > 0.70 for all measurements, *p* < 0.01). Bland–Altman plots displayed significant, systematic bias for Fit3D Proscanner (Redwood City, CA, USA) (all sites: +1.5 cm–4.0 cm), Size Stream SS20 (Cary, NC, USA) (hip and arms +1.6–2.5 cm, thigh −3.0 cm) and Styku S100 (Styku, Los Angeles, CA, USA) (heterogeneous magnitude).	In the processed scans, mean 3DO-tape circumference differences tended to be small (~1–9%) and varied across systems; correlations and bias estimates also varied in strength across anatomic sites and systems. Overall findings differed across devices; the best results were found for the multi-camera stationary system and less so for two rotating single- or dual-camera systems.
Pepper et al. (2011) [32]	Reliability: coefficient of variation (CV), intraclass correlation coefficient (ICC) with 2-way mixed-effects ANOVA Accuracy: standard error of the estimate (SEE), Pearson’s correlation coefficient (r), coefficient of determination (R^2^) of univariate regression analysis, Bland–Altman Plot	CVs showed little difference between within-subject measurements, with a high level of concordance among 8 repeated measures (CV 0.53–1.68%). All ICCs were ≥0.99, with abdomen, waist and hip showing the highest values (ICC = 1.00) and chest circumference having the lowest ICC = 0.992.	No significant differences for waist, hip, or waist:hip ratio according to Paired samples *t*-tests (*p* = 0.05); significant correlation by Pearson’s r (0.998, 0.989, and 0.984 for waist, hip, and waist:hip ratio respectively, *p* = 0.01). No significant systematic bias in Bland–Altman plots shown by r and regression analysis. No impact of age, BMI, and body size on circumference measurement bias in univariate regression analysis	Body volume estimations by laser body scanner and hydrodensitometry were strongly related, and agreement was high. Measurements of % body fat also agreed strongly with each other between methods, and mean % fat estimates by body imaging did not differ from criterion methods. Body imaging is an accurate measure of body fat compared with dual energy X-ray absorptiometry
Sobhiyeh, Kennedy et al. (2021) [43]	Accuracy: mean difference, *t*-test, coefficient of determination (R^2^) of linear regression, Bland–Altman plot, root mean square error (RMSE) in regression analysis		Mean circumference values by CA and DA were comparable.Statistically significant differences were observed (absolute mean ∆ ~2 cm across digital scanners and body sites, with a few outliers). Mean systematic differences were negative for Styku S100 scanner (Los Angeles, CA, USA) and positive for Fit3D Proscanner (Redwood City, CA, USA) and Size Stream SS20 (Cary, NC, USA). Relative CA-DA differences were smaller for chest, waist, and hip measurements (∼2–3%) but larger for arms (∼5–7%) and ankles (∼8–10%). Linear regression analysis showed a RMSE of 1–3 cm, with a trend for higher error for Styku; high R^2^ values were also seen (majority > 0.90, *p* < 0.001), with a few exceptions for limbs. Bland–Altman plots displayed significant systematic bias in 11/33 evaluations; correlations between CA and DA waist circumference estimates had R^2^s of 0.95–0.97 (*p* < 0.001), with measurement bias significant only for the Fit3D Proscanner (Redwood City, CA, USA) (*p* < 0.05).	Site location error sometimes had a significant impact on various girth measurements. The magnitude of this error varied according to the girth measurement being taken, sex, and BMI. Special care should be applied when measuring girths on females, especially waist girths on lean females.
Simenko et al. (2016) [50]	Reliability: coefficient of variation (CV), standard error of measurement (SEM), intraclass correlation coefficient (ICC) Accuracy: Pearson’s correlation coefficient, coefficient of determination (R^2^) of univariate linear regression, Bland–Altman plot, mean differences, *t*-test	DA body circumferences %CV 6.62–11.29, SEM 0.13–0.46 (corresponding manual %CVs and SEMs consistently higher at each body site). Accuracy: 10 out of 14 paired measurements showed statistically significant (*p* < 0.05) but clinically small differences (Mean differences 0.273–0.974 cm; average relative error 0.006–0.037) with non-significant Bland–Altman plots. High correlation and explained variance in univariate linear regression in all measurements (Pearson’s r > 0.96, R^2^ > 0.906).		Digital body scan measurements correlated strongly to criterion methods. However, systematic differences were observed for each measure due to discrepancies in landmark positioning. Predictive body composition equations showed strong agreement for whole body and arms, legs and trunk. Visceral fat prediction showed moderate agreement.

Abbreviations: CA = conventional anthropometry; HC = Head circumference; MUAC = Mid-Upper Arm Circumference; WHR = Waist Hip Ratio; WHtR = Waist to Height Ratio; BMI = body mass index; LoA = limits of agreement; Δ = difference; 3DO = 3D optical.

**Table 4:** Statistical analysis of included studies evaluating both classic anthropometric measurements and body composition.

Authors and Year of Publication	Summary Statistics	Results		Conclusion
		Reliability	Accuracy	
Bourgeois et al. (2017) [34]	Reliability: coefficient of variation (CV) Accuracy: coefficient of determination (R^2^), root mean square error (RMSE), mean difference (groups), paired *t*-test, Bland–Altman plot	Comparing the DA and reference method, CV values were lower for CA (between 0.2 and 0.4%) than for DA (between 0.1 and 2.6%), except for the hip circumference with Styku S100 scanner (Styku, Los Angeles, CA, USA) (0.2% in CA, 0.1% in DA), and lower with DXA (between 0.2 and 1.5%) than with DA (between 0.4 and 5.7%), except with the Styku S100 scanner (Los Angeles, CA, USA) for the trunk (0.6% with DXA, 0.3% with the scan) and left leg (1% with DXA, 0.8% with scan).	Measures obtained with DA significantly correlated with CA (R^2^ 0.72–0.96, *p* < 0.0001, RMSE 1.9–7.7), DXA (R^2^ 0.69–0.99, *p* < 0.0001, RMSE 0.8–12) and ADP (R^2^ 0.99, *p* < 0.0001). Significant difference of means between DA and CA, except for waist with KX-16 scanner (TC LABS, Aoex, NC, USA), hip and right thigh with Styku S100 scanner (Styku, Los Angeles, CA, USA) and right arm with Fit3D Proscanner (Redwood City, CA, USA), and between DA and ADP and DXA. Bland–Altman plots showed a significant underestimation, especially for subjects with higher volumes, except for hip circumference and trunk volume, with R^2^ ranging from 0.0005 to 0.85.	The reliability was higher in the reference methods (tape measurements and DXA). The measurements of circumferences and regional body volume obtained from 3D optical devices were well correlated with those obtained from tape measurements and DXA, but there were significant differences and an underestimation, especially in body volume for larger subjects; total body volume determined by DA were highly correlated with ADP volumes.
Wong et al. (2019) [37]	Reliability: coefficient of variation (CV) Accuracy: coefficient of determination (R^2^), root mean squared error (RMSE), Bland–Altman plot	In CA, %CVs of circumferences (waist: 0.28%, hip: 0.20%, arm: 0.46%, thigh: 0.98%) were lower than CVs in DA (waist: 1.37%, hip: 0.79%, arm: 2.51%, thigh: 2.59%); %CVs of indices of body shape in DA were 1.50% for waist-hip ratio, 1.82% for waist-height ratio and 1.29% for waist-width ratio; %CVs of measures of body composition in DA were 3.30% for FM and 1.34% for FFM.	Strong association between DA and CA for waist circumference (R^2^ = 0.939, RMSE 3.783) and hip (R^2^ = 0.987, RMSE 1.828) and between DA and DXA (total body volume: R^2^ 0.995, RMSE 1.618; trunk volume: R^2^ 0.968, RMSE 1.683; arm volume: R^2^ 0.968, RMSE 0.255; leg volume: R^2^ 0.763, RMSE 2.159; %FM: R^2^ 0.855, RMSE 3.630). Bland–Altman plot showed a good agreement for %BF and a size related bias for waist and hip circumferences and regional body volumes.	Each method was reliable and estimates of 3D body composition and circumferences were strongly associated with the manual measurements and DXA. With the strong correlation and low RMSE, 3D can substitute as a reasonable alternative method if DXA is not available. There was an overestimation of waist and hip circumferences and the volumes for total body, leg and arm and an underestimation for trunk volume.
Kennedy et al. (2020) [39]	Reliability: coefficient of variation (%CV), mean difference (among repeated measures) Accuracy: paired *t*-test, coefficient of determination (R^2^) of multiple regression, Bland–Altman plot, root mean square error (RMSE) in regression analysis, mean group differences (between methods)	Naked Body Scanners (Naked Labs Inc., Redwood City, CA, USA) showed lower repeatability than CA for body circumferences (CV 0.4–2.7% vs. 0.2–0.4%). The average mean difference between duplicate measurements by DA was 0.4 ± 0.4 cm for hip and 0.7 ± 0.7 cm for waist circumferences (CA: 0.2 ± 0.4 cm for all locations). The most precise measurement was hip circumference (DA:CV = 0.4%; CA:CV = 0.2%). %BF was consistent among repeated measures by DA (CV = 2.4%).	Mean group differences between DA and CA ranged from 1.5 cm (arms) and 3.2 cm (thighs). Only hip circumference was not significantly different between the two methods. For all sites, explained variance in linear regression by DA was high (R^2^s, 0.84–0.97; *p* < 0.0001). Bland–Altman plots displayed how the Naked Body Scanners (Naked Labs Inc., Redwood City, CA, USA) significantly overestimated waist circumferences by ~2.0 cm compared with CA (*p* < 0.0001). Significant bias was also found for left and right thighs, with a mean overestiamtion of ~3.0 cm (*p* < 0.0001). %BF: no significant difference between DA and DXA, with a linear regression R^2^ = 0.73 (*p* < 0.0001). Bland–Altman plot revealed a quasi-significant systematic bias by DA to underestimate %BF (*p* = 0.09).	DA exhibited greater variation in test–retest reliability between the six measured anatomic locations compared with manual measurements. All six device-derived circumferences correlated with flexible tape references. The %fat estimates correlated with DXA results with no significant bias.
Pepper et al. (2010) [31]	Reliability: coefficient of variation (CV), intraclass correlation coefficient (ICC) Accuracy: Pearson’s correlation coefficient (r), coefficient of determination (R^2^) of univariate linear regression, standard error of the estimate (SEE), ICC (between methods)	Volumes: all ICCs were ≥0.99, with thigh volume having the lowest ICC (0.997). CVs showed little difference between within-subject measurements, with total body volume the most reliable (CV 0.41%) and thigh volume the most variable (CV 2.26%).	%BF showed good agreement among all methods, with an overall ICC = 0.86 (*p* < 0.01); RM-ANOVA showed no significant difference after pairwise comparison between DA and DXA or hydrodensitometry mean %BF (*p* = 0.81 and 0.43, respectively).	Evaluation of waist and hip circumferences measured by body scanner did not differ significantly from tape measure (*p* < 0.05), with no bias between laser scanning and tape measure. The 3-dimensional laser body scanner is reliable and valid in order to estimate waist and hip circumference compared with tape measure.
Ng et al. (2016) [45]	Reliability: coefficient of variation (CV). Accuracy: mean difference, paired *t*-test, coefficient of determination (R^2^) of stepwise multiple regression, root mean square error (RMSE)	Good test-retest precision with %CV < 5% in almost all measurements: 0.75–2.24% (circumferences), 0.81–3.45% (surface area), volume (0.91–4.49%). Higher CV% in derived regional FM and FFM (VAT: 6.69%; Arms FFM: 6.67%; Arms FM: 11.63%). RMSE 0.03–1.41 (generally 0.5–0.7).	For calibration group, significant paired *t*-test for waist and hip circumferences. Mean differences were 1.75 cm (CI 0.58–2.91) and 3.17 cm (CI 1.43–4.41), respectively. High association with ADP and DXA whole body volumes were observed (R^2^ = 0.99 and 0.97, respectively). Regional DA volume estimates showed strong correlation with DXA measured counterparts (R^2^ = 0.73–0.97), with less volume included in limbs and relatively more volume in the trunk compartment (all *p* < 0.001).	The R^2^ of the prediction equations for fat mass and percent body fat were 93.2% and 76.4% for android, and 91.4% and 66.5% for gynoid, respectively. Prediction values for fat mass and percent fat were 94.6% and 88.9% for total body, 93.9% and 71.0% for trunk, and 92.4% and 64.1% for leg, respectively
Wang et al. (2006) [46]	Reliability: coefficient of variation (CV), intraclass correlation coefficient (ICC). Accuracy: paired *t*-test, Pearson’s correlation coefficient, coefficient of determination (R^2^), standard error of the estimate (SEE), mean absolute differences	Lowest CVs for circumferences (0.9) and partial thigh length (1.2). As for volumes, the CVs ranged from 1.9 to 2.5 for head, upper and lower limbs and torso volumes, while total-body volume showed a CV = 0.4. All ICCs > 0.97 (lenghts, volumes, circumferences).	No significant difference in %BF according to paired *t*-tests between 3DPS and UWW (*p* = 0.4801), although differences were higher than in volumes. Total body volumes between 3DPS and UWW showed high linear correlation with R^2^ = 0.999 and SEE = 0.892 L, with DA showing significant slightly greater values than UWW (*p* < 0.001). Body circumferences values by DA were slightly greater than CA ones (*p* < 0.001).	Body shape of white American adults differs from that of their UK counterparts. Among Americans, ethnic differences in body shape closely track reported differences in prevalence of metabolic syndrome. 3D photonic scanning offers a novel approach for categorizing the risk of metabolic syndrome.
Tinsley, Benavides et al. (2020) [47]	Reliability: RMS-% CV (=%TEM), coefficient of variation (CV), intraclass correlation coefficient (ICC) with 2-way mixed-effects ANOVA. Accuracy: coefficient of determination (R^2^) of univariate linear regression, Bland–Altman plot, root mean square error (RMSE) in regression analysis	Body circumferences: all body scanners showed high precision with ICCs 0.974–0.999. When averaged across all body regions, the four body scanners produced a RMS-% CV of 1.1–1.3%, with the lowest value for hip (RMS-% CV < 1% for all), followed by waist (0.7–1.6%) and thigh (0.8–1.4%), neck (1.2–2.0%), and arm circumferences (1.4–2.8%). Three scanners measured body volumes, with ICCs 0.952–0.999. When averaged across all body regions, RMS-% CV was 1.9–2.3%, with the lowest value for total body (RMS-% CV < 1% for all), followed by torso (~1.2%), leg (~2.5%), and arm (~3 to 5%) volumes.	Total BV: strong linear correlation was observed between DA and reference methods (R^2^: 0.98–1.0); significant overestimation by Size Stream^®^ (Cary, NC, USA) and underestimation by Styku S100 scanner^®^ (Los Angeles, CA, USA) was observed (*p* < 0.01) and no true equivalence from Fit3D Proscanner^®^ (Redwood City, CA, USA) (in contrast to all DXA-derived equations). Bland–Altman plots showed systematic proportional bias of various degrees for all four scanners. DA RMSE: 4.2–10.5 L, with LoA 2.9–5.3 L (both larger compared with DXA-related indices). Similar accuracy issues (strong linear correlation with significant overestimation and proportional bias) were reported in regional volumes.	All scanners produced precise estimates. Precision for circumferences generally decreased in the order of: hip, waist and thigh, chest, neck, and arms. Precision for volumes generally decreased in the order of: BV, torso, legs, and arms. No total or regional 3DO volume estimates exhibited equivalence with reference methods using 5% equivalence regions, and proportional bias of varying magnitudes was observed.

Abbreviations: VAT = Visceral Adipose Tissue; RM = Repeated measures; 3DPS = three-dimensional photonic scan; UWW = underwater weighing; BV = body volume; L = liter.

**Table 5:** Recap of the statistical analysis of included studies.

Study	Random Error **Precision (P)** **Absolute Reliability (AR)** **Relative Reliability (RR)** Precision (Reliability): Precision and Reliability Are Used Synonymously							Systematic Error or Bias Accuracy or Validity Accuracy (Validity): Accuracy and Validity Are Used Synonymously							
	P			AR		RR		Correlation, at a Mean Level							Agreement or Concordance at an Individual Level
	TEM	%TEM	PE	CV	SEM	R	ICC	r	R^2^	SEE	RMSE	CCC	Δ Means	*t*-Test	Bland–Altman Plot
Harbin et al. (2018) [33]								X					X	X	X
Garlie et al. (2010) [26]								X		X		X	X	X	X
Sobhiyeh, Kennedy et al. (2021) [43]									X		X		X	X	X
Tinsley, Benavides et al. (2020) [47]		X	X				X		X		X		X	X	X
Kennedy et al. (2022) [42]									X				X	X	X
Tinsley, Adamson et al. (2020) [41]		X	X				X				X	X	X		X
Pepper et al. (2010) [31]				X			X	X	X	X			X	X	X
Pepper et al. (2011) [32]				X			X	X	X				X		X
Lee et al. (2015) [44]									X	X					X
Wang et al. (2006) [46]				X			X		X	X	X		X	X	
Bourgeois et al. (2017) [34]				X					X		X		X	X	X
Wagner et al. (2019) [35]					X		X	X	X	X			X	X	X
Cabre et al. (2021) [36]								X	X	X	X		X	X	X
Wong et al. (2019) [44]				X					X		X				X
Conkle et al. (2018) [38]	X	X				X	X								X
Heuberger et al. (2008) [29]									X						
Kennedy et al. (2020) [39]				X					X		X		X	X	X
Sobhiyeh, Dunkel et al. (2021) [40]									X				X		
Koepke et al. (2017) [24]	X						X	X				X	X	X	X
Sager et al. (2020) [25]													X		X
Beckmann et al. (2019) [27]								X				X	X	X	X
Wells et al. (2015) [48]	X							X	X	X			X	X	X
Busic et al. (2020) [49]				X	X			X	X				X	X	X
Simenko et al. (2016) [50]				X	X		X	X	X				X	X	X
Japar et al. (2017) [28]													X	X	
Milanese et al. (2015) [30] *								X	X	X			X	X	
Lu et al. (2010) [51]													X	X	
Ng et al. (2016) [45]				X					X		X		X	X	

* Accuracy values were not reported in Supplementary Materials Tables S4–S7 since this study did not evaluate digital anthropometry’s ability to predict baseline body composition. Actually, Milanese et al. [30] compared exercise-induced regional changes in body FM (as measured by DXA) with changes in selected body geometrical measures (as measured by a digital scanner). See Table 6 for more details. Abbreviations: PE = precision error; R = coefficient of reliability.

**Table 6:** Statistical analysis of included studies evaluating body composition, volume, FM and FFM.

Authors and Year of Publication	Summary Statistics	Results		Conclusion
		Reliability	Accuracy	
Harbin et al. (2018) [33]	Accuracy: Pearson’s coefficient (r), mean difference (groups), Bland–Altmnn plot		The correlation between DA and other methods for %BF was measured: r was 0.816 with HW, 0.888 with BIA, 0.817 with skinfolds, and 0.875 with circumferences. According to Bland–Altman plot, there was a proportional bias when measuring %BF in DA compared with circumferences (mean differences: −3.20 ± 9.022, LoA: −12.22–5.822), skinfolds (mean differences: −1.743 ± 1.133, LoA: −12.88–9.391), BIA (mean differences: −1.954 ± 8.167, LoA: −10.12–6.213), HW (mean differences: −4.704 ± 9.808, LoA: −14.51–5.104) and a reduced accuracy among subjects with increased adiposity.	The %BF from 3D was underestimated compared with other methods, and there was a proportional bias, probably attributable to inconsistencies with landmark and partition position in the 3D scan analysis algorithm.
Wagner et al. (2019) [35]	Reliability: standard error of measurement (SEM), intraclass correlation coefficient (ICC) Accuracy: Pearson’s coefficient (r), coefficient of determination (R^2^), standard error of estimate (SEE), mean difference (groups), *t*-test, Bland–Altman plot	The ICC and the SEM for the DA was 0.995 and 0.57, showing a high test-retest reliability.	There was a high correlation between DA and ADP for %BF (r = 0.899, r^2^ = 0.809, SEE 4.13%), but the mean difference (mean DA: 24 ± 6.8%; mean ADP: 21.9 ± 9.4%) and Bland–Altman (r = −0.597, LoA −6.7 to 11%) showed a significant (*p* < 0.001) and proportional bias with an overestimation of the lean body.	The scanner overestimated participants at the lean end of the sample and underestimated participants with the most body fat, not providing valid estimates of %BF compared with ADP.
Garlie et al. (2010) [26]	Accuracy: Pearson’s coefficient (r), concordance correlation coefficient (CCC), standard error of estimate (SEE), mean difference (groups), paired *t*-test, Bland–Altman plot		%BF measured by DA was correlated with %BF measured by DXA (r = 0.74, SEE 3.2, *p* < 0.05) and by CA (r = 0.96, SEE 1, *p* < 0.05). CCC revealed a moderate and statistically significant concordance correlation between DA and DXA (rho_c: 0.74) and DA and CA (rho_c: 0.96). There were no significant differences between DA and CA for height, neck and waist circumferences or between DA and DXA for %BF (mean differences: 0.11% ± 3.1%, LoA: −6.06–6.28%).	The correlation and concordance were high with DA and there were no significant differences of means.
Cabre et al. (2021) [36]	Accuracy: Pearson’s coefficient (r), coefficient of determination (R^2^), standard error of estimate (SEE), root mean square error (RMSE), mean difference (groups), *t*-test, Bland–Altman plot		Strong correlation between 3D and DXA and 4C (for DXA: %BF r = 0.86, R^2^ = 0.74; %BF with 4C, r = 0.80, R^2^ = 0.63; FM with 4C FM, r = 0.9, R^2^ = 0.81; FFM r = 0.9, R^2^ = 0.88; for 4C: %BF, r = 0.80, R^2^ = 0.63; FM, r = 0.85, R^2^ = 0.72; FFM, r = 0.92, R^2^ = 0.84), and SEE was fairly good in %BF with DXA (4.20%), good in FM with DXA (2.91 kg), fair in FFM with DXA (3.77 kg) and FM with 4C (3.64 kg), poor in %BF and FFM with 4C (5.31% and 4.76 kg, respectively). The differences between DA and DXA were −0.10% for %BF, −0.28 kg for FM and −0.10 kg for FFM. Differences between DA and 4C were higher: 4.13% for %BF, 2.66 kg for FM, −3.15 kg for FF. According to paired *t*-test and Bland– Altman plot, there were significant differences between DA and 4C, not for values between DA and DXA but the LoA was wide (%BF: −8.46–8.25; FM: −5.99–5.42; FFM: −7.68–7.48).	DA produced acceptable measurements compared with DXA, and the two methods were in good agreement, especially in those with normal or high lean mass, but the LoA was wide so the agreement should be interpreted with caution. DA does not appear to be valid against 4C models.
Sobhiyeh, Dunkel et al. (2021) [40]	Accuracy: one-way ANOVA, Pearson’s correlation coefficient (r), coefficient of determination (R^2^) of stepwise multiple regression, mean absolute error (MAE)		Volumes: total body volume measured by DXA and ADP showed high linear correlation with DA using a universal software (R^2^ 0.98, MAE 1.34–2.17 for Styku S100 scanner® (Styku, Los Angeles, CA, USA); R^2^ = 1.00, MAE 1.19–1.79 for SS20 Size Stream SS20^®^ (Cary, NC, USA)); a slight significant underestimation by Styku was observed. As for regional volumes, good agreement between DA and DXA was seen the regional volumes calculated by DXA (arms: R^2^ 0.75 vs. 0.79, legs: R^2^ 0.86 vs. 0.89, trunk: R^2^ 0.97 vs. 0. 98, for Styku S100 scanner® (Styku, Los Angeles, CA, USA) vs. SS20 Size Stream SS20^®^ (Cary, NC, USA). However, a significant though positive bias in the head-neck region was observed, with R^2^ 0.43–0.59. Fat mass (FM) calculated with Siri equation: total FM by DA strongly agreed with DXA counterpart (R^2^ = 0.84 vs. 0.86, Styku S100 scanner® (Styku, Los Angeles, CA, USA) vs. SS20 Size Stream SS20^®^ (Cary, NC, USA). Appendicular fat masses estimated by the universal software also showed good agreement with DXA regional fat masses (R^2^ 0.72–0.88 for Styku S100 scanner® (Styku, Los Angeles, CA, USA) and R^2^ 0.76–0.85 for SS20 Size Stream SS20^®^ (Cary, NC, USA)), which were still lower than ADP values.	Total body and regional volumes measured by DXA and ADP had strong associations with corresponding estimates from the commercial 3D optical scanners coupled with the universal software. Regional body volumes also had strong correlation between DXA and the 3DO scanners. Similarly, there were strong associations between DXA-measured total body and regional fat mass and 3D optical estimates calculated by the universal software. Absolute differences in volumes and fat mass between the reference methods and the universal software values appeared.
Tinsley, Adamson et al. (2020) [41]	Reliability: relative technical error of measurement (%TEM = RMS-% CV), precision error (PE), intraclass correlation coefficient (ICC) with 2-way mixed-effects ANOVA. Accuracy: mean difference, Bland–Altman plot, concordance correlation coefficient (CCC), root mean square error (RMSE) in regression analysis	All body scanners showed ICCs 0.975–0.999 (*p* < 0.0001). Naked 3D Fitness Trackers^®^ (Redwood City, CA, USA), Styku S100 scanner® (Styku, Los Angeles, CA, USA), and Size Stream SS20^®^ (Cary, NC, USA) were the most precise in terms of %BF (PE 0.5–0.7%, RMS-% CV 2.3–2.9%), with FIT3D Proscanner^®^ (Redwood City, CA, USA)^®^ showing slightly higher errors (PE: 1.0–1.1%; CV: 4.0–4.3%). All scanners showed similar precision for FM (kg), while FFM exhibited a RMS-% CV 0.7–1.4% for all scanners.	Size Stream SS20^®^ (Cary, NC, USA), FIT3D Proscanner^®^ (Redwood City, CA, USA), and Naked 3D Fitness Trackers^®^ (Redwood City, CA, USA) were equivalent to the 4C model in terms of %BF, FM, and FFM (5% equivalence region = ±1.3% body fat, ±1.0 kg FM, and ±2.7 kg FFM). All scanners CCCs: 0.74–0.90 (%BF), 0.85–0.95 (FM), 0.93–0.97 (FFM). FIT3D Proscanner^®^ (Redwood City, CA, USA) displayed the lowest RMSE for all variables (2.8 kg for FM and FFM; 3.7% for %BF), similar to Naked 3D Fitness Trackers^®^ (Redwood City, CA, USA) and Size Stream SS20^®^ (Cary, NC, USA); Styku S100 scanner® (Styku, Los Angeles, CA, USA) displayed the largest RMSE (4.6 kg for FM and FFM, 6.1% for %BF). Bland–Altman plots showed that FIT3D Proscanner^®^ (Redwood City, CA, USA) had narrowest LoA (±7% for %BF and ±~5.5 kg for FM and FFM), with slightly higher values for all other scanners (±~9.0–9.5% for %BF and ±~7.0 kg for FM and FFM). Proportional bias was largest for %BF, with regression coefficients ± 0.1–0.3 for all scanners (*p* < 0.01). Naked 3D Fitness Trackers^®^ (Redwood City, CA, USA) showed no systematic bias for FM, and Styku S100 scanner® (Styku, Los Angeles, CA, USA) showed no systematic bias for FFM.	All scanners produced reasonably reliable estimates and, except Styku, demonstrated equivalence with 4C, using 5% equivalence regions, and constant errors of <1% for %BF and 0.5 kg for FM and FFM.
Milanese et al. (2015) [30]	Accuracy: paired *t*-test, Pearson’s correlation coefficient (r), coefficient of determination (R^2^) of multiple linear regression, standard error of the estimate (SEE)		All pre-post absolute changes in DA whole-body FM showed fair linear correlation with DXA counterparts (r > 0.5); 4 out of 6 regional DA trunk FM changes correlated with DXA measurements. As for relative changes, only TB %FM and trunk % FM correlated with their respective DA measurements. When individually used as predictor variables in simple linear regression analysis, several DA anthropometric measurements produced significant models (*p* < 0.05, adjusted R^2^ 12.0–39.9%) with no improvement when implemented in a stepwise regression analysis.	Variation in DXA-measured FM and % FM (at both the TB and trunk level) of women with obesity after exercise training showed several significant correlations, with variation in automatic digital anthropometric measurements.
Lee et al. (2015) [44]	Accuracy: coefficient of determination (R^2^) of stepwise multiple regression, standard error of the estimate (SEE), mean errors, Bland–Altman plot		Prediction equations for android and gynoid FM (kg) showed higher prediction values (R^2^ 93.2% android and 91.4% gynoid) than %BF (76.4% android and 66.5% gynoid). Total and regional body FM (kg) was better predicted (R^2^ from 92.4 to 94.6%), as opposed to predicted %BF (R^2^ from 64.1 to 89.9%). Cross-validation of the proposed equations showed no statistical difference between DXA and predicted body fat by the equation (all mean error CI included 0). Android and gynoid FM and %BF data distribued within Bland–Altman plots, 95% LoA with few outliers and a systematic bias ~0 cm.	Overall, group mean digital and conventional body circumferences values were in good agreement, with ∼2 cm systematic differences and highly correlated (all *p* values < 0.01). The bias tended to be small, but significant depending on anatomic site and device.

Abbreviations: FM = Fat Mass; FFM = Free Fat Mass; BF = body fat; CI = confidence interval; 4C = 4-component model; rho_c = Lin’s concordance correlation; TB = total body.

**Table 7:** GRADE assessment for overall quality of evidence.

Outcomes	Risk of Bias	Inconsistency	Indirectness	Imprecision	Publication Bias	Overall Quality of Evidence
Anthropometric measures	Serious	Not serious	Not serious	Serious	Not serious	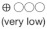
Body composition	Serious	Not serious	Serious	Serious	Not serious	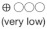

## Data Availability

No new data were created or analyzed in this study. Data sharing is not applicable to this article.

## Supplementary Materials

The following supporting information can be downloaded at: https://www.mdpi.com/article/10.3390/nu15020302/s1, Table S1: PRISMA 2020 Checklist [18]; Table S2: Results of the quality assessment of the included studies through Newcastle–Ottawa Scale for cross-sectional studies [24,25,26,27,28,29,30,31,32,33,34,35,36,37,38,39,40,41,42,43,44,45,46,47,48,49,50,51]; Table S3: Results of the quality assessment of the included studies through the AXIS tool [24,25,26,27,28,29,30,31,32,33,34,35,36,37,38,39,40,41,42,43,44,45,46,47,48,49,50,51]; Table S4: Statistical analysis of included studies evaluating the random error in classic anthropometric measurements: circumferences and lengths [24,32,34,37,38,39,45,46,47,48,49,50]; Table S5: Statistical analysis of included studies evaluating the systematic error in classic anthropometric measurements: circumferences and lengths [24,25,27,28,29,32,34,37,38,39,42,43,45,46,48,49,50,51]; Table S6: Statistical analysis of included studies evaluating the random error in body composition, volume, FM and FFM [31,34,35,37,39,41,45,46]; Table S7: Statistical analysis of included studies evaluating the systematic error in body composition, volume, FM and FFM [26,31,33,34,35,36,37,39,40,41,44,45,46,47].
