# Supplementary material for: Digital Anthropometry: A Systematic Review on Precision, Reliability and Accuracy of Most Popular Existing Technologies
Source: Nutrients. 2023 Jan 7;15(2):302. doi: 10.3390/nu15020302 (PMC9864001; doi:10.3390/nu15020302)
Supplement: Supplementary file 1 [file nutrients-15-00302-s001.zip › nutrients-2082242-supplementary.pdf]

Table S1. PRISMA 2020 Checklist [18].

Section and Topic	Item #	Checklist item	Reported on page #
**TITLE**			
Title	1	Identify the report as a systematic review.	1
**ABSTRACT**			
Abstract	2	See the PRISMA 2020 for Abstracts checklist.	
**INTRODUCTION**			
Rationale	3	Describe the rationale for the review in the context of existing knowledge.	1
Objectives	4	Provide an explicit statement of the objective(s) or question(s) the review addresses.	2
**METHODS**			
Eligibility criteria	5	Specify the inclusion and exclusion criteria for the review and how studies were grouped for the syntheses.	4
Information sources	6	Specify all databases, registers, websites, organisations, reference lists and other sources searched or consulted to identify studies. Specify the date when each source was last searched or consulted.	4
Search strategy	7	Present the full search strategies for all databases, registers and websites, including any filters and limits used.	4
Selection process	8	Specify the methods used to decide whether a study met the inclusion criteria of the review, including how many reviewers screened each record and each report retrieved, whether they worked independently, and if applicable, details of automation tools used in the process.	4/5
Data collection process	9	Specify the methods used to collect data from reports, including how many reviewers collected data from each report, whether they worked independently, any processes for obtaining or confirming data from study investigators, and if applicable, details of automation tools used in the process.	5
Data items	10a	List and define all outcomes for which data were sought. Specify whether all results that were compatible with each outcome domain in each study were sought (e.g., for all measures, time points, analyses), and if not, the methods used to decide which results to collect.	4
	10b	List and define all other variables for which data were sought (e.g., participant and intervention characteristics, funding sources). Describe any assumptions made about any missing or unclear information.	4
Study risk of bias assessment	11	Specify the methods used to assess risk of bias in the included studies, including details of the tool(s) used, how many reviewers assessed each study and whether they worked independently, and if applicable, details of automation tools used in the process.	5
Effect measures	12	Specify for each outcome the effect measure(s) (e.g., risk ratio, mean difference) used in the synthesis or presentation of results.	
Synthesis methods	13a	Describe the processes used to decide which studies were eligible for each synthesis (e.g., tabulating the study intervention characteristics and comparing against the planned groups for each synthesis (item #5)).	5
	13b	Describe any methods required to prepare the data for presentation or synthesis, such as handling of missing summary statistics, or data	5

Section and Topic	Item #	Checklist item	Reported on page #
		conversions.	
	13c	Describe any methods used to tabulate or visually display results of individual studies and syntheses.	5
	13d	Describe any methods used to synthesize results and provide a rationale for the choice(s). If meta-analysis was performed, describe the model(s), method(s) to identify the presence and extent of statistical heterogeneity, and software package(s) used.	5
	13e	Describe any methods used to explore possible causes of heterogeneity among study results (e.g., subgroup analysis, meta-regression).	5
	13f	Describe any sensitivity analyses conducted to assess robustness of the synthesized results.	5
Reporting bias assessment	14	Describe any methods used to assess risk of bias due to missing results in a synthesis (arising from reporting biases).	5
Certainty assessment	15	Describe any methods used to assess certainty (or confidence) in the body of evidence for an outcome.	5
**RESULTS**			
Study selection	16a	Describe the results of the search and selection process, from the number of records identified in the search to the number of studies included in the review, ideally using a flow diagram.	5
	16b	Cite studies that might appear to meet the inclusion criteria, but which were excluded, and explain why they were excluded.	7/8
Study characteristics	17	Cite each included study and present its characteristics.	9
Risk of bias in studies	18	Present assessments of risk of bias for each included study.	9
Results of individual studies	19	For all outcomes, present, for each study: (a) summary statistics for each group (where appropriate) and (b) an effect estimate and its precision (e.g. confidence/credible interval), ideally using structured tables or plots.	17–23
Results of syntheses	20a	For each synthesis, briefly summarise the characteristics and risk of bias among contributing studies.	9
	20b	Present results of all statistical syntheses conducted. If meta-analysis was done, present for each the summary estimate and its precision (e.g. confidence/credible interval) and measures of statistical heterogeneity. If comparing groups, describe the direction of the effect.	9–15
	20c	Present results of all investigations of possible causes of heterogeneity among study results.	9–15
	20d	Present results of all sensitivity analyses conducted to assess the robustness of the synthesized results.	9–15
Reporting biases	21	Present assessments of risk of bias due to missing results (arising from reporting biases) for each synthesis assessed.	9
Certainty of evidence	22	Present assessments of certainty (or confidence) in the body of evidence for each outcome assessed.	26
**DISCUSSION**			

Section and Topic	Item #	Checklist item	Reported on page #
Discussion	23a	Provide a general interpretation of the results in the context of other evidence.	26/27
	23b	Discuss any limitations of the evidence included in the review.	26/27
	23c	Discuss any limitations of the review processes used.	26/27
	23d	Discuss implications of the results for practice, policy, and future research.	26/27
**OTHER INFORMATION**			
Registration and protocol	24a	Provide registration information for the review, including register name and registration number, or state that the review was not registered.	
	24b	Indicate where the review protocol can be accessed, or state that a protocol was not prepared.	
	24c	Describe and explain any amendments to information provided at registration or in the protocol.	
Support	25	Describe sources of financial or non-financial support for the review, and the role of the funders or sponsors in the review.	
Competing interests	26	Declare any competing interests of review authors.	
Availability of data, code and other materials	27	Report which of the following are publicly available and where they can be found: template data collection forms; data extracted from included studies; data used for all analyses; analytic code; any other materials used in the review.	

From: [18] Page, M.J., McKenzie, J.E.; Bossuyt, P.M.; Boutron, I.; Hoffmann, T.C.; Mulrow, C.D.; Shamseer, L.; Tetzlaf, J.M.; Akl, E.A.; Brennan, S.E.; et al. The PRISMA 2020 statement: an updated guideline for reporting systematic reviews. *BMJ* **2021**, *372*, 71. https://doi.org/10.1136/bmj.n71.

Table S2. Results of the quality assessment of the included studies through NewCastle Ottawa Scale for cross sectional studies [24–51].

Authors and Year of Publication	Selection				Comparability	Outcome		Total Rating
	Representativeness of the Sample	Sample Size	Non-Respondents	Ascertainment of Exposure	Based on Design and Analysis	Assessment of Outcome	Statistical Test	
Harbin et al. (2018) [33]	*			*		**	*	5
Bourgeois et al. (2017) [34]	*	*		*		**	*	6
Koepke et al. (2017) [24]	*			*		**	*	5
Garlie et al. (2010) [26]	*			*		**	*	5
Wagner et al. (2019) [35]	*			*		**	*	5
Busic et al. (2020) [49]				*		**	*	4
Sager et al. (2020) [25]	*			*		**	*	5
Japar et al. (2017) [28]	*			*		**	*	5
Cabre et al. (2021) [36]	*			*		**	*	5
Wells et al. (2015) [48]	*			*		**	*	5
Wong et al. (2019) [37]	*			*		**	*	5
Conkle et al. (2018) [38]	*			*		**	*	5
Heuberger et al. (2008) [29]	*			*		**	*	5
Lu et al. (2010) [51]				*		**	*	4
Beckmann et al. (2019) [27]		*		*		**	*	5
Kennedy et al. (2020) [39]	*			*		**	*	5
Sobhiyeh, Dunkel et al. (2021) [40]	*	*		*		**	*	6
Tinsley, Benavides et al. (2020) [47]	*	*		*		**	*	6
Kennedy et al. (2021) [42]	*	*		*		**	*	6
Tinsley, Adamson et al. (2020) [41]	*	*		*		**	*	6
Milanese et al. (2015) [30]	*			*		**	*	5
Pepper et al. (2010) [31]	*			*		**	*	5
Pepper et al. (2011) [32]	*			*		**	*	5
Sobhiyeh, Kennedy et al. (2021) [43]	*	*		*		**	*	6
Lee et al. (2015) [44]	*	*		*		**	*	6
Ng et al. (2016) [45]	*	*		*		**	*	6
Simenko et al. (2016) [50]	*			*		**	*	5
Wang et al. (2006) [46]	*			*		**	*	5

Abbreviations: * = 1 point; ** = 2 points.

Table S3. Results of the quality assessment of the included studies through AXIS tool [24–51].

Authors and Year of Publication	Questions																				Total Rating
	1	2	3	4	5	6	7	8	9	10	11	12	13	14	15	16	17	18	19	20	
Harbin et al. (2018) [33]	Y	Y	N	Y	Y	Y	NA	Y	Y	N	Y	Y	NA	NA	Y	N	Y	Y	N	Y	13
Bourgeois et al. (2017) [34]	Y	Y	Y	Y	Y	Y	NA	Y	Y	Y	Y	Y	NA	NA	Y	N	Y	Y	N	Y	15
Koepke et al. (2017) [24]	Y	Y	N	Y	Y	Y	NA	Y	Y	Y	Y	Y	NA	NA	Y	Y	Y	Y	N	Y	15
Garlie et al. (2010) [26]	Y	Y	N	Y	Y	Y	NA	Y	Y	Y	Y	Y	NA	NA	Y	Y	Y	Y	Y	Y	16
Wagner et al. (2019) [35]	Y	Y	N	Y	N	Y	NA	Y	Y	Y	Y	Y	NA	NA	Y	N	Y	N	Y	Y	13
Busic et al. (2020) [49]	Y	Y	N	N	DNK	DNK	NA	Y	Y	Y	Y	N	NA	NA	Y	N	Y	Y	Y	Y	11
Sager et al. (2020) [25]	Y	Y	N	Y	N	Y	NA	Y	Y	Y	Y	Y	NA	NA	Y	Y	Y	Y	Y	Y	15
Japar et al. (2017) [28]	Y	Y	N	Y	N	N	NA	Y	Y	Y	N	Y	NA	NA	Y	Y	Y	N	N	Y	11
Cabre et al. (2021) [36]	Y	Y	N	Y	Y	Y	NA	Y	Y	Y	Y	Y	NA	NA	Y	Y	Y	Y	N	Y	15
Wells et al. (2015) [48]	Y	Y	N	Y	Y	N	NA	Y	Y	Y	Y	Y	NA	NA	Y	Y	Y	N	Y	Y	14
Wong et al. (2019) [37]	Y	Y	N	Y	Y	N	NA	Y	Y	Y	Y	Y	NA	NA	Y	N	Y	Y	Y	Y	14
Conkle et al. (2018) [38]	Y	Y	N	Y	Y	Y	NA	Y	Y	Y	Y	Y	NA	NA	Y	Y	Y	Y	Y	Y	16
Heuberger et al. (2008) [29]	Y	Y	N	Y	Y	Y	NA	Y	Y	Y	Y	Y	NA	NA	Y	Y	Y	Y	Y	DNK	15
Lu et al. (2010) [51]	Y	Y	N	N	DNK	DNK	NA	Y	Y	N	Y	Y	NA	NA	Y	Y	Y	N	DNK	DNK	9
Beckmann et al. (2019) [27]	Y	Y	Y	N	N	N	NA	Y	Y	Y	Y	Y	NA	NA	Y	Y	Y	Y	N	Y	13
Kennedy et al. (2020) [39]	Y	Y	N	Y	Y	Y	NA	Y	Y	Y	Y	Y	NA	NA	Y	Y	Y	Y	N	Y	15
Sobhiyeh, Dunkel et al. (2021) [40]	Y	Y	Y	Y	Y	Y	NA	Y	Y	Y	Y	Y	NA	NA	Y	Y	Y	Y	N	Y	16
Tinsley, Benavides et al. (2020) [47]	Y	Y	Y	Y	Y	Y	NA	Y	Y	Y	Y	Y	NA	NA	Y	Y	Y	Y	N	Y	16
Kennedy et al. (2021) [42]	Y	Y	Y	Y	Y	Y	NA	Y	Y	Y	Y	Y	NA	NA	Y	Y	Y	Y	N	Y	16
Tinsley, Adamson et al. (2020) [41]	Y	Y	Y	Y	Y	Y	NA	Y	Y	Y	Y	Y	NA	NA	Y	Y	Y	Y	N	Y	16
Milanese et al. (2015) [30]	Y	Y	N	Y	Y	Y	NA	Y	Y	Y	Y	Y	NA	NA	Y	Y	Y	N	N	Y	14
Pepper et al. (2010) [31]	Y	Y	N	Y	Y	Y	NA	Y	Y	Y	Y	Y	NA	NA	Y	Y	Y	Y	N	Y	15
Pepper et al. (2011) [32]	Y	Y	N	Y	Y	Y	NA	Y	Y	Y	Y	Y	NA	NA	Y	Y	Y	Y	N	Y	15
Sobhiyeh, Kennedy et al. (2021) [43]	Y	Y	Y	Y	Y	Y	NA	Y	Y	Y	Y	Y	NA	NA	Y	Y	Y	Y	N	Y	16
Lee et al. (2015) [44]	Y	Y	Y	Y	Y	Y	NA	Y	Y	Y	Y	Y	NA	NA	Y	Y	Y	Y	N	Y	16
Ng et al. (2016) [45]	Y	Y	Y	Y	Y	Y	NA	Y	Y	Y	Y	Y	NA	NA	Y	Y	Y	Y	N	Y	16
Simenko et al. (2016) [50]	Y	Y	N	Y	DNK	DNK	NA	Y	Y	Y	Y	Y	NA	NA	Y	Y	Y	Y	N	Y	13
Wang et al. (2006) [46]	Y	Y	N	Y	DNK	DNK	NA	Y	N	Y	Y	Y	NA	NA	Y	Y	Y	Y	N	Y	12

Abbreviations: Y = yes; N = no; DNK = not known; NA = not applicable.

Table S4. Statistical analysis of included studies evaluating the random error in classic anthropometric measurements: circumferences and lengths [24,32,34,37–39,45–50] *.

Circumferences	Random Error<br>• Precision (P)<br>• Absolute Reliability (AR)<br>• Relative Reliability (RR)<br>Precision (reliability): Precision and reliability are used synonymously						
	P			AR		RR	
	TEM	%TEM	PE	%CV	SEM	R	ICC
Height	0.45 Koepke et al. (2017) [24]<br>0.51 Conkle et al. (2018) [38]			5.26 Busic et al. (2020) [49]	0.51 Busic et al. (2020) [49]	1.0 Conkle et al. (2018) [38]	1.0 Koepke et al. (2017) [24]<br>1.0 Conkle et al. (2018) [38]
Head	0.33 Conkle et al. (2018) [38]					0.99 Conkle et al. (2018) [38]	1.0 Conkle et al. (2018) [38]
Waist	0.98 Koepke et al. (2017) [24]<br>1.49 Wells et al. (2015) [48]	1.2 Tinsley, Benavides et al. (2020) [17]<br>1.1 Tinsley, Benavides et al. (2020) [47]<br>0.9 Tinsley, Benavides et al. (2020) [47]<br>1.1 Tinsley, Benavides et al. (2020) [47]	1.0 Tinsley, Benavides et al. (2020) [47]<br>1.0 Tinsley, Benavides et al. (2020) [47]<br>0.9 Tinsley, Benavides et al. (2020) [47]<br>0.6 Tinsley, Benavides et al. (2020) [47]	7.14 Busic et al. (2020) [49]<br>0.8 Bourgeois et al. (2017) [34]<br>0.3 Bourgeois et al. (2017) [34]<br>1.37 Wong et al. (2019) [37]<br>0.83 Pepper et al. (2011) [32]<br>0.7 Kennedy et al. (2020) [39]<br>1.5 Ng et al. (2016) [45]<br>0.88 Wang et al. (2006) [46]	0.61 Busic et al. (2020) [49]		0.99 Koepke et al. (2017) [24]<br>0.99 Tinsley, Benavides et al. (2020) [47]<br>0.99 Tinsley, Benavides et al. (2020) [47]<br>0.99 Tinsley, Benavides et al. (2020) [47]<br>1.0 Tinsley, Benavides et al. (2020) [47]<br>1.0 Pepper et al. (2011) [32]<br>0.999 Wang et al. (2006) [46]

Hip	2.50 Koepke et al. (2017) [24]	0.8 Tinsley, Benavides et al. (2020) [47]<br>0.6 Tinsley, Benavides et al. (2020) [47]<br>0.4 Tinsley, Benavides et al. (2020) [47]<br>0.5 Tinsley, Benavides et al. (2020) [47]	0.8 Tinsley, Benavides et al. (2020) [47]<br>0.5 Tinsley, Benavides et al. (2020) [47]<br>0.4 Tinsley, Benavides et al. (2020) [47]<br>0.5 Tinsley, Benavides et al. (2020) [47]	4.24 Busic et al. (2020) [49]<br>0.4 Bourgeois et al. (2017) [34]<br>0.1 Bourgeois et al. (2017) [34]<br>0.79 Wong et al. (2019) [37]<br>0.53 Pepper et al. (2011) [32]<br>0.4 Kennedy et al. (2020) [39]<br>0.75 Ng et al. (2016) [45]<br>0.5 Wang et al. (2006) [46]	0.85 Busic et al. (2020) [49]	0.97 Koepke et al. (2017) [24]<br>0.99 Tinsley, Benavides et al. (2020) [47]<br>0.99 Tinsley, Benavides et al. (2020) [47]<br>0.99 Tinsley, Benavides et al. (2020) [47]<br>0.99 Tinsley, Benavides et al. (2020) [47]<br>1.0 Pepper et al. (2011) [32]<br>0.999 Wang et al. (2006) [46]
Breast				7.69 Busic et al. (2020) [49]	0.92 Busic et al. (2020) [49]	
Chest	1.24 Koepke et al. (2017) [24]<br>0.13 Wells et al. (2015) [48]	0.7 Tinsley, Benavides et al. (2020) [47]<br>1.1 Tinsley, Benavides et al. (2020) [47]<br>0.9 Tinsley, Benavides et al. (2020) [47]<br>1.7 Tinsley, Benavides et al. (2020) [47]	0.7 Tinsley, Benavides et al. (2020) [47]<br>1.1 Tinsley, Benavides et al. (2020) [47]<br>0.9 Tinsley, Benavides et al. (2020) [47]<br>1.6 Tinsley, Benavides et al. (2020) [47]	7.52 Busic et al. (2020) [49]<br>1.34 Pepper et al. (2011) [32]<br>0.81 Wang et al. (2006) [46]	1.40 Busic et al. (2020) [49]	0.97 Koepke et al. (2017) [24]<br>0.99 Tinsley, Benavides et al. (2020) [47]<br>0.99 Tinsley, Benavides et al. (2020) [47]<br>0.99 Tinsley, Benavides et al. (2020) [47]<br>0.98 Tinsley, Benavides et al. (2020) [47]<br>0.992 Pepper et al. (2011) [32]<br>0.998 Wang et al. (2006) [46]
Buttock	1.18 Koepke et al. (2017) [24]					0.95 Koepke et al. (2017) [24]

Right upper arm	0.31 Conkle et al. (2018) [38]	1.6 Tinsley, Benavides et al. (2020) [47]<br>1.4 Tinsley, Benavides et al. (2020) [47]<br>1.7 Tinsley, Benavides et al. (2020) [47]<br>2.7 Tinsley, Benavides et al. (2020) [47]	0.5 Tinsley, Benavides et al. (2020) [47]<br>0.5 Tinsley, Benavides et al. (2020) [47]<br>0.6 Tinsley, Benavides et al. (2020) [47]<br>0.7 Tinsley, Benavides et al. (2020) [47]	9.8 Busic et al. (2020) [49]<br>2.6 Bourgeois et al. (2017) [34]<br>1.2 Bourgeois et al. (2017) [34]<br>0.8 Bourgeois et al. (2017) [34]<br>2.51 Wong et al. (2019) [37]<br>10.89 Simenko et al. (2016) [50]<br>1.4 Kennedy et al. (2020) [39]<br>2.24 Ng et al. (2016) [45]	0.50 Busic et al. (2020) [49]<br>0.46 Simenko et al. (2016) [50]	0.98 Conkle et al. (2018) [38]	0.99 Conkle et al. (2018) [38]<br>0.99 Tinsley, Benavides et al. (2020) [47]<br>0.99 Tinsley, Benavides et al. (2020) [47]<br>0.98 Tinsley, Benavides et al. (2020) [47]<br>0.97 Tinsley, Benavides et al. (2020) [47]
Left upper arm	0.31 Conkle et al. (2018) [38]	1.6 Tinsley, Benavides et al. (2020) [47]<br>1.8 Tinsley, Benavides et al. (2020) [47]<br>2.8 Tinsley, Benavides et al. (2020) [47]<br>1.9 Tinsley, Benavides et al. (2020) [47]	0.5 Tinsley, Benavides et al. (2020) [47]<br>0.6 Tinsley, Benavides et al. (2020) [47]<br>1.0 Tinsley, Benavides et al. (2020) [47]<br>0.5 Tinsley, Benavides et al. (2020) [47]	10.29 Busic et al. (2020) [49]<br>2.51 Wong et al. (2019) [37]<br>11.29 Simenko et al. (2016) [50]<br>2.7 Kennedy et al. (2020) [39]<br>2.24 Ng et al. (2016) [45]	0.45 Busic et al. (2020) [49]<br>0.41 Simenko et al. (2016) [50]	0.98 Conkle et al. (2018) [38]	0.99 Conkle et al. (2018) [38]<br>0.99 Tinsley, Benavides et al. (2020) [47]<br>0.98 Tinsley, Benavides et al. (2020) [47]<br>0.95 Tinsley, Benavides et al. (2020) [47]<br>0.99 Tinsley, Benavides et al. (2020) [47]
Right forearm				9.61 Busic et al. (2020) [49]<br>6.09 Wong et al. (2019) [37]<br>10.63 Simenko et al. (2016) [50]<br>1.93 Ng et al. (2016) [45]	0.34 Busic et al. (2020) [49]<br>0.31 Simenko et al. (2016) [50]		

Left forearm				9.34 Busic et al. (2020) [49]<br>6.09 Wong et al. (2019) [37]<br>11.11 Simenko et al. (2016) [50]<br>1.93 Ng et al. (2019) [38]	0.29 Busic et al. (2020) [49]<br>0.20 Simenko et al. (2016) [50]		
Right wrist				8.48 Busic et al. (2020) [49]<br>7.62 Simenko et al. (2016) [50]	0.32 Busic et al. (2020) [49]<br>0.17 Simenko et al. (2016) [50]		
Left wrist				7.77 Busic et al. (2020) [49]<br>7.98 Simenko et al. (2016) [50]	0.32 Busic et al. (2020) [49]<br>0.19 Simenko et al. (2016) [50]		
Right thigh		0.9 Tinsley, Benavides et al. (2020) [47]<br>1.2 Tinsley, Benavides et al. (2020) [47]<br>1.0 Tinsley, Benavides et al. (2020) [47]<br>0.9 Tinsley, Benavides et al. (2020) [47]	0.5 Tinsley, Benavides et al. (2020) [47]<br>0.7 Tinsley, Benavides et al. (2020) [47]<br>0.6 Tinsley, Benavides et al. (2020) [47]<br>0.6 Tinsley, Benavides et al. (2020) [47]	6.05 Busic et al. (2020) [49]<br>0.9 Bourgeois et al. (2017) [34]<br>0.7 Bourgeois et al. (2017) [34]<br>0.3 Bourgeois et al. (2017) [34]<br>2.59 Wong et al. (2019) [37]<br>1.10 Pepper et al. (2011) [32]<br>7.3 Simenko et al. (2016) [50]<br>1.2 Kennedy et al. (2020) [39]<br>0.95 Ng et al. (2016) [45]	0.56 Busic et al. (2020) [49]<br>0.23 Simenko et al. (2016) [50]		0.99 Tinsley, Benavides et al. (2020) [47]<br>0.98 Tinsley, Benavides et al. (2020) [47]<br>0.99 Tinsley, Benavides et al. (2020) [47]<br>0.99 Tinsley, Benavides et al. (2020) [47]<br>0.999 Pepper et al. (2011) [32]<br>1.0 Wang et al. (2006) [46]

				0.36 Wang et al. (2006) [46]			
Left thigh		0.9 Tinsley, Benavides et al. (2020) [47]<br>1.4 Tinsley, Benavides et al. (2020) [47]<br>0.8 Tinsley, Benavides et al. (2020) [47]<br>1.0 Tinsley, Benavides et al. (2020) [47]	0.5 Tinsley, Benavides et al. (2020) [47]<br>0.8 Tinsley, Benavides et al. (2020) [47]<br>0.5 Tinsley, Benavides et al. (2020) [47]<br>0.6 Tinsley, Benavides et al. (2020) [47]	7.03 Busic et al. (2020) [49]<br>2.59 Wong et al. (2019) [37]<br>1.10 Pepper et al. (2011) [32]<br>7.72 Simenko et al. (2016) [50]<br>1.1 Kennedy et al. (2020) [39]<br>0.95 Ng et al. (2016) [45]<br>0.36 Wang et al. (2006) [46]	0.43 Busic et al. (2020) [49]<br>0.35 Simenko et al. (2016) [50]		0.99 Tinsley, Benavides et al. (2020) [47]<br>0.97 Tinsley, Benavides et al. (2020) [47]<br>0.99 Tinsley, Benavides et al. (2020) [47]<br>0.99 Tinsley, Benavides et al. (2020) [47]<br>0.999 Pepper et al. (2011) [32]<br>1.0 Wang et al. (2006) [46]
Right knee	1.36 Wells et al. (2015) [48]			1.59 Pepper et al. (2011) [32]<br>6.65 Simenko et al. (2016) [50]<br>0.5 Wang et al. (2006) [46]	0.40 Simenko et al. (2016) [50]		0.998 Pepper et al. (2011) [32]<br>0.999 Wang et al. (2006) [46]
Left knee	1.36 Wells et al. (2015) [48]			1.59 Pepper et al. (2011) [32]<br>6.62 Simenko et al. (2016) [50]<br>0.5 Wang et al. (2006) [46]	0.35 Simenko et al. (2016) [50]		0.998 Pepper et al. (2011) [32]<br>0.999 Wang et al. (2006) [46]
Right calf	0.90 Wells et al. (2015) [48]			5.95 Busic et al. (2020) [49]<br>1.56 Wong et al. (2019) [37]<br>1.03 Pepper et al. (2011) [32]	0.59 Busic et al. (2020) [49]<br>0.17 Simenko et al. (2016) [50]		0.999 Pepper et al. (2011) [32]

			8.12 Simenko et al. (2016) [50]<br>0.92 Ng et al. (2016) [45]			
Left calf	0.90 Wells et al. (2015) [48]		7.39 Busic et al. (2020) [49]<br>1.56 Wong et al. (2019) [37]<br>1.03 Pepper et al. (2011) [32]<br>7.64 Simenko et al. (2016) [50]<br>0.92 Ng et al. (2016) [45]	0.62 Busic et al. (2020) [49]<br>0.13 Simenko et al. (2016) [50]		0.999 Pepper et al. (2011) [32]

* When multiple values of the same reliability index (e.g., TEM) are displayed for the same study, each value refers to a different digital scanner (commercial name not reported). Abbreviations: TEM = absolute Technical Error of Measurement; %TEM = relative Technical Error of Measurement; PE = precision error; %CV = coefficient of variation; SEM = standard error of estimate; R = coefficient of reliability; ICC = intraclass correlation coefficient.

Table S5. Statistical analysis of included studies evaluating the systematic error in classic anthropometric measurements: circumferences and lengths [24,25,27–29,32,34,37–39,42,43,45,46,48–51] *.

Circumferences	Systematic Error or Bias<br>Accuracy or Validity<br>Accuracy (validity): Validity and accuracy are used synonymously						
	**Correlation, at a Mean Level**						**Agreement or Concordance, at an Individual Level**
	**r**	**R²**	**SEE**	**RMSE**	**CCC**	**Mean Difference (*p* value)**	**Bland-Altman Plot: 95% LoA** (Lower Level, Upper Level)
Height	0.99 Busic et al. (2020) [49]<br>0.99 Beckmann et al. (2019) [27]<br>0.99 Koepke et al. (2017) [24]	0.99 Busic et al. (2020) [49]			0.99 Beckmann et al. (2019) [27]<br>0.95 Koepke et al. (2017) [24]	−0.49 (0.007) Busic et al. (2020) [49]<br>−2.01 (<0.001) Koepke et al. (2017) [24]<br>−0.77 (<0.001) Beckmann et al. (2019) [27]<br>−1 Sager et al. (2020) [25]<br>5.76 (0.47) Lu et al. (2010) [51]	−3.52, −0.50 Koepke et al. (2017) [24]<br>−2.4, 1.4 Busic et al. (2020) [49]
Head							−0.26, 1.37 Conkle et al. (2018) [38]<br>−0.62, 1.14 Conkle et al. (2018) [38]
Waist	0.97 Busic et al. (2020) [49]<br>0.98 Beckmann et al. (2019) [27]<br>0.98 Koepke et al. (2017) [24]	0.95 Busic et al. (2020) [49]<br>0.86 Bourgeois et al. (2017) [34]<br>0.92 Bourgeois et al. (2017) [34]	2.37 Wells et al. (2015) [48]	5.3 Bourgeois et al. (2017) [34]<br>5.8 Bourgeois et al. (2017) [34]<br>6.3 Bourgeois et al. (2017) [34]	0.96 Beckmann et al. (2019) [27]<br>0.96 Koepke et al. (2017) [24]	−1.69 (0.00) Busic et al. (2020) [49]<br>0.64 (<0.05) Bourgeois et al. (2017) [34]<br>4.21 (>0.05) Bourgeois et al. (2017) [34]	−0.92, 1.43 Koepke et al. (2017) [24]<br>−0.7, 4.1 Busic et al. (2020) [49]<br>−3.29, 6.01 Wells et al. (2015) [48]

	0.96 Wells et al. (2015) [48]<br>0.998 Pepper et al. (2011) [32]	0.94 Bourgeois et al. (2017) [34]<br>0.92 Wells et al. (2015) [48]<br>0.94 Wong et al. (2019) [37]<br>0.96 Kennedy et al. (2021) [36]<br>0.95 Kennedy et al. (2021) [36]<br>0.93 Kennedy et al. (2021) [36]<br>0.995 Pepper et al. (2011) [32]<br>0.96 Kennedy et al. (2020) [39]<br>0.95 Ng et al. (2016) [45]<br>0.95 Sobhiyeh, Kennedy et al. (2021) [43]<br>0.97 Sobhiyeh, Kennedy et al. (2021) [43]<br>0.97 Sobhiyeh, Kennedy et al. (2021) [43]		3.78 Wong et al. (2019) [37]<br>3.16 Kennedy et al. (2020) [39]<br>3.12 Ng et al. (2016) [45]<br>3.27 Sobhiyeh, Kennedy et al. (2021) [43]<br>2.54 Sobhiyeh, Kennedy et al. (2021) [43]<br>2.60 Sobhiyeh, Kennedy et al. (2021) [43]		−5.08 (<0.01) Bourgeois et al. (2017) [34]<br>1.5 (<0.0001) Beckmann et al. (2019) [27]<br>1.17 (<0.001) Koepke et al. (2017) [24]<br>0.71 (<0.05) Japar et al. (2017) [28]<br>1.36 (<0.001) Wells et al. (2015) [48]<br>14.73 (0.009) Lu et al. (2010) [51]<br>1.5 (<0.001) Kennedy et al. (2021) [36]<br>1.6 (<0.001) Kennedy et al. (2021) [36]<br>−5.5 (<0.001) Kennedy et al. (2021) [36]<br>0.13 (>0.005) Pepper et al. (2011) [32]<br>1.7 (<0.001) Kennedy et al. (2020) [39]<br>1.75 (<0.005) Ng et al. (2016) [45]<br>11.4 (<0.01) Wang et al. (2006) [46]<br>−5.7 (<0.001) Sobhiyeh, Kennedy et al. (2021) [43]<br>2.5 (<0.001) Sobhiyeh, Kennedy et al. (2021) [43]<br>2.9 (<0.001) Sobhiyeh, Kennedy et al. (2021) [43]	
Hip	0.92 Busic et al. (2020) [49]	0.85 Busic et al. (2020) [49]		6.0 Bourgeois et al. (2017) [34]	0.72 Koepke et al. (2017) [24]	1.37 (0.00) Busic et al. (2020) [49]	4.01, 4.74 Koepke et al. (2017) [24]

	0.93 Koepke et al. (2017) [24]<br>0.989 Pepper et al. (2011) [32]	0.91 Bourgeois et al. (2017) [34]<br>0.90 Bourgeois et al. (2017) [34]<br>0.96 Bourgeois et al. (2017) [34]<br>0.99 Wong et al. (2019) [37]<br>0.63 Heuberger et al. (2008) [29]<br>0.95 Kennedy et al. (2021) [36]<br>0.97 Kennedy et al. (2021) [36]<br>0.97 Kennedy et al. (2021) [36]<br>0.978 Pepper et al. (2011) [32]<br>0.97 Kennedy et al. (2020) [39]<br>0.92 Ng et al. (2016) [45]<br>0.95 Sobhiyeh, Kennedy et al. (2021) [43]<br>0.97 Sobhiyeh, Kennedy et al. (2021) [43]<br>0.97 Sobhiyeh, Kennedy et al. (2021) [43]	4.6 Bourgeois et al. (2017) [34]<br>2.6 Bourgeois et al. (2017) [34]<br>1.83 Wong et al. (2019) [37]<br>2.01 Kennedy et al. (2020) [39]<br>3.48 Ng et al. (2016) [45]<br>1.93 Sobhiyeh, Kennedy et al. (2021) [43]<br>1.31 Sobhiyeh, Kennedy et al. (2021) [43]<br>1.34 Sobhiyeh, Kennedy et al. (2021) [43]	4.8 (>0.05) Bourgeois et al. (2017) [34]<br>1.2 (<0.01) Bourgeois et al. (2017) [34]<br>−0.02 (<0.0001) Bourgeois et al. (2017) [34]<br>4.37 (<0.001) Koepke et al. (2017) [24]<br>8.59 (<0.01) Japar et al. (2017) [28]<br>10.23 (0.60) Lu et al. (2010) [51]<br>4.2 (<0.001) Kennedy et al. (2021) [36]<br>1.7 (<0.001) Kennedy et al. (2021) [36]<br>1.0 (<0.001) Kennedy et al. (2021) [36]<br>−0.24 Pepper et al. (2011) [32]<br>−0.2 (>0.005) Kennedy et al. (2020) [39]<br>3.17 (<0.005) Ng et al. (2016) [45]<br>19.0 (<0.01) Wang et al. (2006) [46]<br>−2.9 (<0.001) Sobhiyeh, Kennedy et al. (2021) [43]<br>1.3 (<0.001) Sobhiyeh, Kennedy et al. (2021) [43]<br>1.9 (<0.001) Sobhiyeh, Kennedy et al. (2021) [43]	−1.9, 4.6 Busic et al. (2020) [49]
Breast	0.97 Busic et al. (2020) [49]	0.94 Busic et al. (2020) [49]		1.80 (0.05) Busic et al. (2020) [49]	−2.9, 4.2 Busic et al. (2020) [49]

Chest	0.94 Busic et al. (2020) [49]<br>0.94 Koepke et al. (2017) [24]<br>0.97 Wells et al. (2015) [48]	0.88 Busic et al. (2020) [49]<br>0.94 Wells et al. (2015) [48]<br>0.99 Wang et al. (2006) [46]<br>0.94 Sobhiyeh, Kennedy et al. (2021) [43]<br>0.97 Sobhiyeh, Kennedy et al. (2021) [43]<br>0.95 Sobhiyeh, Kennedy et al. (2021) [43]	2.16 Wells et al. (2015) [48]<br>21.06 Wang et al. (2006) [46]	2.31 Sobhiyeh, Kennedy et al. (2021) [43]<br>1.64 Sobhiyeh, Kennedy et al. (2021) [43]<br>2.49 Sobhiyeh, Kennedy et al. (2021) [43]	0.78 Koepke et al. (2017) [24]	3.93 (0.00) Busic et al. (2020) [49]<br>3.88 (<0.001) Koepke et al. (2017) [24]<br>3.67 (<0.001) Wells et al. (2015) [48]<br>(0.0008) Lu et al. (2010) [51]<br>15.9 (<0.01) Wang et al. (2006) [46]<br>−0.6 (>0.005) Sobhiyeh, Kennedy et al. (2021) [43]<br>3.0 (<0.001) Sobhiyeh, Kennedy et al. (2021) [43]<br>5.2 (<0.001) Sobhiyeh, Kennedy et al. (2021) [43]	−0.37, 8.13 Koepke et al. (2017) [24]<br>−1.8, 9.6 Busic et al. (2020) [49]<br>−0.70, 8.04 Wells et al. (2015) [48]
Buttock	0.83 Koepke et al. (2017) [24]				0.26 Koepke et al. (2017) [24]	12.62 (<0.001) Koepke et al. (2017) [24]	15.93, 17.31 Koepke et al. (2017) [24]
Right upper arm	0.94 Busic et al. (2020) [49]<br>0.97 Simenko et al. (2016) [50]	0.89 Busic et al. (2020) [49]<br>0.75 Bourgeois et al. (2017) [34]<br>0.87 Bourgeois et al. (2017) [34]<br>0.73 Bourgeois et al. (2017) [34]<br>0.75 Kennedy et al. (2021) [36]<br>0.73 Kennedy et al. (2021) [36]<br>0.72 Kennedy et al. (2021) [36]<br>0.97 Simenko et al. (2016) [50]		3.9 Bourgeois et al. (2017) [34]<br>1.9 Bourgeois et al. (2017) [34]<br>5.0 Bourgeois et al. (2017) [34]<br>1.32 Kennedy et al. (2020) [39]<br>1.37 Sobhiyeh, Kennedy et al. (2021) [43]<br>1.05 Sobhiyeh, Kennedy et al. (2021) [43]		1.28 (0.00) Busic et al. (2020) [49]<br>2.8 (<0.05) Bourgeois et al. (2017) [34]<br>0.04 (>0.05) Bourgeois et al. (2017) [34]<br>−4.1 (<0.0001) Bourgeois et al. (2017) [34]<br>1.8 (<0.001) Kennedy et al. (2021) [36]<br>1.9 (<0.001) Kennedy et al. (2021) [36]<br>−1.7 (<0.001) Kennedy et al. (2021) [36]<br>0.97 (<0.001) Simenko et al. (2016) [50]	−0.63, 3.19 Busic et al. (2020) [49]<br>−1.15, 0.27 Conkle et al. (2018) [38]<br>−0.89, 0.61 Conkle et al. (2018) [38]<br>−1.07, 2.74 Simenko et al. (2016) [50]

		0.95 Kennedy et al. (2020) [39]<br>0.92 Sobhiyeh, Kennedy et al. (2021) [43]<br>0.95 Sobhiyeh, Kennedy et al. (2021) [43]<br>0.96 Sobhiyeh, Kennedy et al. (2021) [43]	1.19 Sobhiyeh, Kennedy et al. (2021) [43]	1.5 (<0.001) Kennedy et al. (2020) [39]<br>−3.5 (<0.001) Sobhiyeh, Kennedy et al. (2021) [43]<br>1.0 (<0.001) Sobhiyeh, Kennedy et al. (2021) [43]<br>3.1 (<0.001) Sobhiyeh, Kennedy et al. (2021) [43]	
Left upper arm	0.96 Busic et al. (2020) [49]<br>0.98 Simenko et al. (2016) [50]	0.92 Busic et al. (2020) [49]<br>0.70 Kennedy et al. (2021) [36]<br>0.68 Kennedy et al. (2021) [36]<br>0.54 Kennedy et al. (2021) [36]<br>0.85 Simenko et al. (2016) [50]<br>0.95 Kennedy et al. (2020) [39]<br>0.93 Sobhiyeh, Kennedy et al. (2021) [43]<br>0.92 Sobhiyeh, Kennedy et al. (2021) [43]<br>0.97 Sobhiyeh, Kennedy et al. (2021) [43]	1.2 Kennedy et al. (2020) [39]<br>1.30 Sobhiyeh, Kennedy et al. (2021) [43]<br>1.44 Sobhiyeh, Kennedy et al. (2021) [43]<br>0.99 Sobhiyeh, Kennedy et al. (2021) [43]	1.45 (0.00) Busic et al. (2020) [49]<br>1.4 (<0.001) Kennedy et al. (2021) [36]<br>1.5 (<0.001) Kennedy et al. (2021) [36]<br>−1.5 (<0.001) Kennedy et al. (2021) [36]<br>0.85 (<0.001) Simenko et al. (2016) [50]<br>1.7 (<0.001) Kennedy et al. (2020) [39]<br>−2.9 (<0.001) Sobhiyeh, Kennedy et al. (2021) [43]<br>2.4 (<0.001) Sobhiyeh, Kennedy et al. (2021) [43]<br>3.2 (<0.001) Sobhiyeh, Kennedy et al. (2021) [43]	22.2, 30.1 Busic et al. (2020) [49]<br>−1.15, 0.27 Conkle et al. (2018) [38]<br>−0.89, 0.61 Conkle et al. (2018) [38]<br>−0.86, 2.48 Simenko et al. (2016) [50]
Right forearm	0.96 Busic et al. (2020) [49]	0.93 Busic et al. (2020) [49]		−0.49 (0.00) Busic et al. (2020) [49]	-1.81, 0.82 Busic et al. (2020) [49]

	0.98 Simenko et al. (2016) [50]	0.96 Simenko et al. (2016) [50]		−0.60 (<0.001) Simenko et al. (2016) [50]	-1.61, 0.75 Simenko et al. (2016) [50]
Left forearm	0.97 Busic et al. (2020) [49]<br>0.99 Simenko et al. (2016) [50]	0.94 Busic et al. (2020) [49]<br>0.98 Simenko et al. (2016) [50]		−0.4 (0.001) Busic et al. (2020) [49]<br>0.40 (<0.001) Simenko et al. (2016) [50]	-1.56, 0.76 Busic et al. (2020) [49]<br>-1.03, 0.51 Simenko et al. (2016) [50]
Right wrist	0.91 Busic et al. (2020) [49]<br>0.96 Simenko et al. (2016) [50]	0.83 Busic et al. (2020) [49]<br>0.93 Simenko et al. (2016) [50]		0.03 (0.77) Busic et al. (2020) [49]<br>0.35 (0.07) Simenko et al. (2016) [50]	-1.14, 1.21 Busic et al. (2020) [49]<br>-0.56, 0.8 Simenko et al. (2016) [50]
Left wrist	0.90 Busic et al. (2020) [49]<br>0.97 Simenko et al. (2016) [50]	0.79 Busic et al. (2020) [49]<br>0.95 Simenko et al. (2016) [50]		−0.08 (0.47) Busic et al. (2020) [49]<br>0.40 (0.002) Simenko et al. (2016) [50]	-1.28, 1.12 Busic et al. (2020) [49]<br>-0.54, 1.04 Simenko et al. (2016) [50]
Right thigh	0.95 Busic et al. (2020) [49]<br>0.99 Simenko et al. (2016) [50]	0.90 Busic et al. (2020) [49]<br>0.71 Bourgeois et al. (2017) [34]<br>0.79 Bourgeois et al. (2017) [34]<br>0.83 Bourgeois et al. (2017) [34]<br>0.88 Kennedy et al. (2021) [36]<br>0.84 Kennedy et al. (2021) [36]<br>0.81 Kennedy et al. (2021) [36]<br>0.99 Simenko et al. (2016) [50]<br>0.86 Kennedy et al. (2020) [39]	7.7 Bourgeois et al. (2017) [34]<br>6.4 Bourgeois et al. (2017) [34]<br>3.3 Bourgeois et al. (2017) [34]<br>2.26 Kennedy et al. (2020) [39]<br>2.49 Sobhiyeh, Kennedy et al. (2021) [43]<br>2.03 Sobhiyeh, Kennedy et al. (2021) [43]<br>2.82 Sobhiyeh, Kennedy et al. (2021) [43]	−0.47 (0.03) Busic et al. (2020) [49]<br>6.2 (>0.05) Bourgeois et al. (2017) [34]<br>−5.7 (<0.0001) Bourgeois et al. (2017) [34]<br>−0.2 (<0.0001) Bourgeois et al. (2017) [34]<br>−1.2 (<0.001) Kennedy et al. (2021) [36]<br>−3.0 (<0.001) Kennedy et al. (2021) [36]<br>2.9 (<0.001) Kennedy et al. (2021) [36]<br>0.49 (<0.001) Simenko et al. (2016) [50]<br>3.0 (<0.001) Kennedy et al. (2020) [39]<br>4.6 (<0.01) Wang et al. (2006) [46]	−2.7, 1.8 Busic et al. (2020) [49]<br>−0.49, 1.43 Simenko et al. (2016) [50]

		0.77 Sobhiyeh, Kennedy et al. (2021) [43]<br>0.83 Sobhiyeh, Kennedy et al. (2021) [43]<br>0.91 Sobhiyeh, Kennedy et al. (2021) [43]			−2.1 (<0.001) Sobhiyeh, Kennedy et al. (2021) [43]<br>1.1 (<0.005) Sobhiyeh, Kennedy et al. (2021) [43]<br>1.0 (>0.005) Sobhiyeh, Kennedy et al. (2021) [43]	
Left thigh	0.98 Busic et al. (2020) [49]<br>0.99 Simenko et al. (2016) [50]	0.95 Busic et al. (2020) [49]<br>0.82 Kennedy et al. (2021) [36]<br>0.79 Kennedy et al. (2021) [36]<br>0.85 Kennedy et al. (2021) [36]<br>0.98 Simenko et al. (2016) [50]<br>0.84 Kennedy et al. (2020) [39]<br>0.89 Sobhiyeh, Kennedy et al. (2021) [43]<br>0.91 Sobhiyeh, Kennedy et al. (2021) [43]<br>0.92 Sobhiyeh, Kennedy et al. (2021) [43]		2.34 Kennedy et al. (2020) [39]<br>1.78 Sobhiyeh, Kennedy et al. (2021) [43]<br>1.41 Sobhiyeh, Kennedy et al. (2021) [43]<br>1.79 Sobhiyeh, Kennedy et al. (2021) [43]	−0.27 (0.07) Busic et al. (2020) [49]<br>0.3 (>0.005) Kennedy et al. (2021) [36]<br>−3.4 (<0.001) Kennedy et al. (2021) [36]<br>3.8 (<0.001) Kennedy et al. (2021) [36]<br>−0.697 (0.94) Simenko et al. (2016) [50]<br>3.2 (<0.001) Kennedy et al. (2020) [39]<br>4.6 (<0.01) Wang et al. (2006) [46]<br>1.2 (<0.001) Sobhiyeh, Kennedy et al. (2021) [43]<br>2.0 (<0.001) Sobhiyeh, Kennedy et al. (2021) [43]<br>2.2 (<0.001) Sobhiyeh, Kennedy et al. (2021) [43]	−1.89, 1.33 Busic et al. (2020) [49]<br>−1.38, 1.36 Simenko et al. (2016) [50]
Right knee	0.95 Wells et al. (2015) [48]<br>0.95 Simenko et al. (2016) [50]	0.89 Wells et al. (2015) [48]<br>0.91 Simenko et al. (2016) [50]	1.20 Wells et al. (2015) [48]		1.39 (<0.001) Wells et al. (2015) [48]<br>0.79 (<0.001) Simenko et al. (2016) [50]	−0.98, 3.76 Wells et al. (2015) [48]<br>−1.16, 1.95 Simenko et al. (2016) [50]

						14.5 (<0.01) Wang et al. (2006) [46]	
Left knee	0.95 Wells et al. (2015) [48]<br>0.96 Simenko et al. (2016) [50]	0.89 Wells et al. (2015) [48]<br>0.93 Simenko et al. (2016) [50]	1.20 Wells et al. (2015) [48]			1.39 (<0.001) Wells et al. (2015) [48]<br>0.73 (0.98) Simenko et al. (2016) [50]<br>14.5 (<0.01) Wang et al. (2006) [46]	−0.98, 3.76 Wells et al. (2015) [48]<br>−1.43, 1.42 Simenko et al. (2016) [50]
Right calf	0.86 Busic et al. (2020) [49]<br>0.97 Wells et al. (2015) [48]<br>0.99 Simenko et al. (2016) [50]	0.75 Busic et al. (2020) [49]<br>0.95 Wells et al. (2015) [48]<br>0.99 Simenko et al. (2016) [50]<br>0.62 Sobhiyeh, Kennedy et al. (2021) [43]<br>0.97 Sobhiyeh, Kennedy et al. (2021) [43]<br>0.99 Sobhiyeh, Kennedy et al. (2021) [43]	0.8 Wells et al. (2015) [48]	2.04 Sobhiyeh, Kennedy et al. (2021) [43]<br>0.57 Sobhiyeh, Kennedy et al. (2021) [43]<br>0.51 Sobhiyeh, Kennedy et al. (2021) [43]		−0.31 (0.07) Busic et al. (2020) [49]<br>0.62 (<0.001) Wells et al. (2015) [48]<br>0.35 (0.002) Simenko et al. (2016) [50]<br>−3.2 (<0.001) Sobhiyeh, Kennedy et al. (2021) [43]<br>1.5 (<0.001) Sobhiyeh, Kennedy et al. (2021) [43]<br>1.0 (<0.001) Sobhiyeh, Kennedy et al. (2021) [43]	−2.7, 2.0 Busic et al. (2020) [49]<br>−0.95, 2.2 Wells et al. (2015) [48]<br>−0.5, 0.57 Simenko et al. (2016) [50]
Left calf	0.91 Busic et al. (2020) [49]<br>0.97 Wells et al. (2015) [48]<br>1.0 Simenko et al. (2016) [50]	0.83 Busic et al. (2020) [49]<br>0.95 Wells et al. (2015) [48]<br>0.99 Simenko et al. (2016) [50]<br>0.77 Sobhiyeh, Kennedy et al. (2021) [43]	0.8 Wells et al. (2015) [48]	1.41 Sobhiyeh, Kennedy et al. (2021) [43]<br>1.0 Sobhiyeh, Kennedy et al. (2021) [43]<br>0.57 Sobhiyeh, Kennedy et al. (2021) [43]		0.02 (0.94) Busic et al. (2020) [49]<br>0.62 (<0.001) Wells et al. (2015) [48]<br>0.27 (0.47) Simenko et al. (2016) [50]<br>−2.6 (<0.001) Sobhiyeh, Kennedy et al. (2021) [43]<br>2.4 (<0.001) Sobhiyeh, Kennedy et al. (2021) [43]	−2.2, 2.2 Busic et al. (2020) [49]<br>−0.95, 2.2 Wells et al. (2015) [48]<br>−0.89, 0.47 Simenko et al. (2016) [50]

	0.91 Sobhiyeh, Kennedy et al. (2021) [43]<br>0.98 Sobhiyeh, Kennedy et al. (2021) [43]		1.3 (<0.001) Sobhiyeh, Kennedy et al. (2021) [43]

* When multiple values of the same accuracy index (e.g., R^2) are displayed for the same study, each value refers to a different digital scanner (commercial name not reported). Abbreviations: r = Pearson's correlation; R^2 = coefficient of determination; SEE = standard error of the estimate; RMSE = root mean square error; CCC = concordance correlation coefficient; LoA = level of agreement.

Table S6. Statistical analysis of included studies evaluating the random error in body composition, volume, FM and FFM [31,34,35,37,39,41,45,46] *.

**Body Composition**	**Random Error**<br>● **Precision (P)**<br>● **Absolute Reliability (AR)**<br>● **Relative Reliability (RR)**<br><br>Precision (reliability): Precision and eliability are used synonymously				
	**P**		**AR**		**P**
	**%TEM**	**PE**	**%TEM**	**SEM**	**%TEM**
% Body fat	2.5 Tinsley, Adamson et al. (2020) [41]<br>2.3 Tinsley, Adamson et al. (2020) [41]<br>4.0 Tinsley, Adamson et al. (2020) [41]<br>4.3 Tinsley, Adamson et al. (2020) [41]<br>2.9 Tinsley, Adamson et al. (2020) [41]	0.5 Tinsley, Adamson et al. (2020) [41]<br>0.6 Tinsley, Adamson et al. (2020) [41]<br>1.0 Tinsley, Adamson et al. (2020) [41]<br>1.1 Tinsley, Adamson et al. (2020) [41]<br>0.7 Tinsley, Adamson et al. (2020) [41]	2.16 Ng et al. (2016) [45]<br>2.4 Kennedy et al. (2020) [39]	0.57 Wagner et al. (2019) [35]	0.993 Wagner et al. (2019) [35]<br>0.994 Tinsley, Adamson et al. (2020) [41]<br>0.996 Tinsley, Adamson et al. (2020) [41]<br>0.982 Tinsley, Adamson et al. (2020) [41]<br>0.975 Tinsley, Adamson et al. (2020) [41]<br>0.991 Tinsley, Adamson et al. (2020) [41]
Total fat mass	2.6 Tinsley, Adamson et al. (2020) [41]<br>2.5 Tinsley, Adamson et al. (2020) [41]<br>4.3 Tinsley, Adamson et al. (2020) [41]<br>4.0 Tinsley, Adamson et al. (2020) [41]<br>3.1 Tinsley, Adamson et al. (2020) [41]	0.4 Tinsley, Adamson et al. (2020) [41]<br>0.5 Tinsley, Adamson et al. (2020) [41]<br>0.8 Tinsley, Adamson et al. (2020) [41]<br>0.8 Tinsley, Adamson et al. (2020) [41]<br>0.6 Tinsley, Adamson et al. (2020) [41]	3.3 Wong et al. (2019) [37]<br>1.96 Ng et al. (2016) [45]		0.997 Tinsley, Adamson et al. (2020) [41]<br>0.997 Tinsley, Adamson et al. (2020) [41]<br>0.99 Tinsley, Adamson et al. (2020) [41]<br>0.99 Tinsley, Adamson et al. (2020) [41]<br>0.995 Tinsley, Adamson et al. (2020) [41]
Total fat free mass	0.7 Tinsley, Adamson et al. (2020) [41]	0.4 Tinsley, Adamson et al. (2020) [41]	1.34 Wong et al. (2019) [37]<br>0.94 Ng et al. (2016) [45]		0.999 Tinsley, Adamson et al. (2020) [41]

	0.9 Tinsley, Adamson et al. (2020) [41]<br>1.4 Tinsley, Adamson et al. (2020) [41]<br>1.4 Tinsley, Adamson et al. (2020) [41]<br>1.1 Tinsley, Adamson et al. (2020) [41]	0.5 Tinsley, Adamson et al. (2020) [41]<br>0.8 Tinsley, Adamson et al. (2020) [41]<br>0.8 Tinsley, Adamson et al. (2020) [41]<br>0.6 Tinsley, Adamson et al. (2020) [41]		0.999 Tinsley, Adamson et al. (2020) [41]<br>0.996 Tinsley, Adamson et al. (2020) [41]<br>0.996 Tinsley, Adamson et al. (2020) [41]<br>0.998 Tinsley, Adamson et al. (2020) [41]
Visceral fat mass			6.07 Wong et al. (2019) [37]<br>6.69 Ng et al. (2016) [45]	
Body volume	0.6 Tinsley, Benavides et al. (2020) [47]<br>0.8 Tinsley, Benavides et al. (2020) [47]<br>0.9 Tinsley, Benavides et al. (2020) [47]	0.4 Tinsley, Benavides et al. (2020) [47]<br>0.7 Tinsley, Benavides et al. (2020) [47]<br>0.5 Tinsley, Benavides et al. (2020) [47]	0.8 Bourgeois et al. (2017) [34]<br>0.7 Bourgeois et al. (2017) [34]<br>0.4 Bourgeois et al. (2017) [34]<br>1.57 Wong et al. (2019) [37]<br>0.41 Pepper et al. (2010) [31]<br>0.38 Wang et al. (2006) [46]<br>0.74 Ng et al. (2016) [45]	0.999 Tinsley, Benavides et al. (2020) [47]<br>0.998 Tinsley, Benavides et al. (2020) [47]<br>0.998 Tinsley, Benavides et al. (2020) [47]<br>1.0 Pepper et al. (2010) [31]<br>1.0 Wang et al. (2006) [46]
Trunk volume	1.3 Tinsley, Benavides et al. (2020) [47]<br>1.0 Tinsley, Benavides et al. (2020) [47]<br>1.1 Tinsley, Benavides et al. (2020) [47]	0.6 Tinsley, Benavides et al. (2020) [47]<br>0.5 Tinsley, Benavides et al. (2020) [47]<br>0.4 Tinsley, Benavides et al. (2020) [47]	1.0 Bourgeois et al. (2017) [34]<br>0.6 Bourgeois et al. (2017) [34]<br>0.3 Bourgeois et al. (2017) [34]<br>2.43 Wong et al. (2019) [37]<br>1.83 Wang et al. (2006) [46]	0.997 Tinsley, Benavides et al. (2020) [47]<br>0.998 Tinsley, Benavides et al. (2020) [47]<br>0.998 Tinsley, Benavides et al. (2020) [47]<br>0.998 Wang et al. (2006) [46]

			0.99 Ng et al. (2016) [45]	
Right upper limb volume	2.2 Tinsley, Benavides et al. (2020) [47]<br>5.3 Tinsley, Benavides et al. (2020) [47]<br>5.4 Tinsley, Benavides et al. (2020) [47]	0.1 Tinsley, Benavides et al. (2020) [47]<br>0.2 Tinsley, Benavides et al. (2020) [47]<br>0.1 Tinsley, Benavides et al. (2020) [47]	4.1 Bourgeois et al. (2017) [34]<br>2.1 Bourgeois et al. (2017) [34]<br>2.4 Bourgeois et al. (2017) [34]<br>5.46 Wong et al. (2019) [37]<br>2.45 Wang et al. (2006) [46]	0.994 Tinsley, Benavides et al. (2020) [47]<br>0.952 Tinsley, Benavides et al. (2020) [47]<br>0.979 Tinsley, Benavides et al. (2020) [47]<br>0.994 Wang et al. (2006) [46]
Left upper limb volume	3.6 Tinsley, Benavides et al. (2020) [47]<br>4.5 Tinsley, Benavides et al. (2020) [47]<br>4.1 Tinsley, Benavides et al. (2020) [47]	0.1 Tinsley, Benavides et al. (2020) [47]<br>0.2 Tinsley, Benavides et al. (2020) [47]<br>0.1 Tinsley, Benavides et al. (2020) [47]	5.7 Bourgeois et al. (2017) [34]<br>3.9 Bourgeois et al. (2017) [34]<br>2.0 Bourgeois et al. (2017) [34]<br>5.46 Wong et al. (2019) [37]<br>3.10 Wang et al. (2006) [46]	0.983 Tinsley, Benavides et al. (2020) [47]<br>0.957 Tinsley, Benavides et al. (2020) [47]<br>0.986 Tinsley, Benavides et al. (2020) [47]<br>0.991 Wang et al. (2006) [46]
Right lower limb volume	2.6 Tinsley, Benavides et al. (2020) [47]<br>2.5 Tinsley, Benavides et al. (2020) [47]<br>2.6 Tinsley, Benavides et al. (2020) [47]	0.2 Tinsley, Benavides et al. (2020) [47]<br>0.3 Tinsley, Benavides et al. (2020) [47]<br>0.1 Tinsley, Benavides et al. (2020) [47]	1.6 Bourgeois et al. (2017) [34]<br>1.5 Bourgeois et al. (2017) [34]<br>1.4 Bourgeois et al. (2017) [34]<br>5.33 Wong et al. (2019) [37]<br>4.41 Wang et al. (2006) [46]	0.976 Tinsley, Benavides et al. (2020) [47]<br>0.983 Tinsley, Benavides et al. (2020) [47]<br>0.987 Tinsley, Benavides et al. (2020) [47]<br>0.984 Wang et al. (2006) [46]
Left lower limb volume	2.6 Tinsley, Benavides et al. (2020) [47]	0.2 Tinsley, Benavides et al. (2020) [47]	1.6 Bourgeois et al. (2017) [34]	0.974 Tinsley, Benavides et al. (2020) [47]

	2.1 Tinsley, Benavides et al. (2020) [47]<br>2.4 Tinsley, Benavides et al. (2020) [47]	0.2 Tinsley, Benavides et al. (2020) [47]<br>0.1 Tinsley, Benavides et al. (2020) [47]	2.4 Bourgeois et al. (2017) [34]<br>0.8 Bourgeois et al. (2017) [34]<br>5.33 Wong et al. (2019) [37]<br>4.3 Wang et al. (2006) [46]		0.988 Tinsley, Benavides et al. (2020) [47]<br>0.988 Tinsley, Benavides et al. (2020) [47]<br>0.985 Wang et al. (2006) [46]
Right thigh volume			2.26 Pepper et al. (2010) [31]		0.996 Pepper et al. (2010) [31]
Left thigh volume			2.26 Pepper et al. (2010) [31]		0.996 Pepper et al. (2010) [31]

* When multiple values of the same reliability index (e.g., % TEM) are displayed for the same study, each value refers to a different digital scanner (commercial name not reported). Abbreviations: FM = fat mass; FFM = fat free mass.

Table S7. Statistical analysis of included studies evaluating the systematic error in body composition, volume, FM and FFM [26,31,33–37,39–41,44–47] *.

**Body Composition**		**Systematic Error or Bias**						
		**Accuracy or Validity**						
		Accuracy (validity): Validity and accuracy are used synonymously						
		**Correlation, at a Mean Level**						**Agreement or Concordance, at an Individual Level**
	**Reference Method**	**r**	**R^2**	**SEE**	**RMSE**	**CCC**	**Mean Differences (*p* value)**	**Bland-Altman Plot: 95% LoA** (Lower Level, Upper Level)
% Body fat	skinfolds	0.817 Harbin et al. (2018) [33]					−1.74 (<0.001) Harbin et al. (2018) [33]	−12.88, 9.39 Harbin et al. (2018) [33]
	BIA	0.888 Harbin et al. (2018) [33]					−1.95 (<0.001) Harbin et al. (2018) [33]	−10.12, 6.21 Harbin et al. (2018) [33]
	DXA	0.74 Garlie et al. (2010) [26]<br>0.86 Cabre et al. (2021) [36]<br>0.67 Pepper et al. (2010) [31]	0.83 Wong et al. (2019) [37]<br>0.74 Cabre et al. (2021) [36]<br>0.45 Pepper et al. (2010) [31]<br>0.72 Ng et al. (2016) [45]<br>0.73 Kennedy et al. (2020) [39]<br>0.89 Lee et al. (2015) [44]	3.2 Garlie et al. (2010) [26]<br>4.20 Cabre et al. (2021) [36]<br>6.61 Pepper et al. (2010) [31]<br>4.22 Lee et al. (2015) [44]	3.87 Wong et al. (2019) [37]<br>3.75 Ng et al. (2016) [45]	0.74 Garlie et al. (2010) [26]	0.11 (>0.005) Garlie et al. (2010) [26]<br>0.10 (0.74) Cabre et al. (2021) [36]<br>−1.91 (0.43) Pepper et al. (2010) [31]<br>2 (>0.005) Kennedy et al. (2020) [39]	−6.06, 6.28 Garlie et al. (2010) [26]<br>−8.46, 8.25 Cabre et al. (2021) [36]<br>−23.1, 19.27 Pepper et al. (2010) [31]
	ADP	0.899 Wagner et al. (2019) [35]	0.809 Wagner et al. (2019) [35]	4.13 Wagner et al. (2019) [35]			2.1 (<0.001) Wagner et al. (2019) [35]	−6.7, 11.0 Wagner et al. (2019) [35]
	UHWW	0.816 Harbin et al. (2018) [33]	0.47 Pepper et al. (2010) [31]	7.81 Pepper et al. (2010) [31]			−4.70 (<0.001) Harbin et al. (2018) [33]	−14.51, 5.10 Harbin et al. (2018) [33]

		0.69 Pepper et al. (2010) [31]	0.65 Wang et al. (2006) [46]	7.95 Wang et al. (2006) [46]			1.41 (0.81) Pepper et al. (2010) [31]	−19.31, 22.13 Pepper et al. (2010) [31]
	4C model	0.8 Cabre et al. (2021) [36]	0.63 Cabre et al. (2021) [36]	5.31 Cabre et al. (2021) [36]	6.1 Tinsley, Adamson et al. (2020) [41]<br>4.8 Tinsley, Adamson et al. (2020) [41]<br>3.7 Tinsley, Adamson et al. (2020) [41]<br>4.8 Tinsley, Adamson et al. (2020) [41]	0.74 Tinsley, Adamson et al. (2020) [41]<br>0.86 Tinsley, Adamson et al. (2020) [41]<br>0.90 Tinsley, Adamson et al. (2020) [41]<br>0.82 Tinsley, Adamson et al. (2020) [41]	−4.13 (<0.001) Cabre et al. (2021) [36]<br>−4.2 Tinsley, Adamson et al. (2020) [41]<br>−0.4 Tinsley, Adamson et al. (2020) [41]<br>−0.8 Tinsley, Adamson et al. (2020) [41]<br>−0.2 Tinsley, Adamson et al. (2020) [41]	−6.39, 14.69 Cabre et al. (2021) [36]<br>−12.9, 4.5 Tinsley, Adamson et al. (2020) [41]<br>−9.9, 9.1 Tinsley, Adamson et al. (2020) [41]<br>−7.8, 6.2 Tinsley, Adamson et al. (2020) [41]<br>−9.6, 9.2 Tinsley, Adamson et al. (2020) [41]
Total fat mass	DXA	0.90 Cabre et al. (2021) [36]	0.96 Wong et al. (2019) [37]<br>0.81 Cabre et al. (2021) [36]<br>0.86 Sobhiyeh, Dunkel et al. (2021) [40]<br>0.84 Sobhiyeh, Dunkel et al. (2021) [40]<br>0.76 Ng et al. (2016) [45]<br>0.94 Lee et al. (2015) [44]	2.91 Cabre et al. (2021) [36]<br>3.39 Lee et al. (2015) [44]	1.83 Wong et al. (2019) [37]<br>3.72 Ng et al. (2016) [45]		0.28 (0.18) Cabre et al. (2021) [36]<br>0.03 Sobhiyeh, Dunkel et al. (2021) [40]<br>0.09 Sobhiyeh, Dunkel et al. (2021) [40]	−5.99, 5.42 Cabre et al. (2021) [36]
	UHWW						0.72 (0.48) Wang et al. (2006) [46]	

	4C model	0.85 Cabre et al. (2021) [36]	0.72 Cabre et al. (2021) [36]	3.64 Cabre et al. (2021) [36]	4.6 Tinsley, Adamson et al. (2020) [41]<br>3.6 Tinsley, Adamson et al. (2020) [41]<br>2.8 Tinsley, Adamson et al. (2020) [41]<br>3.7 Tinsley, Adamson et al. (2020) [41]	0.85 Tinsley, Adamson et al. (2020) [41]<br>0.92 Tinsley, Adamson et al. (2020) [41]<br>0.95 Tinsley, Adamson et al. (2020) [41]<br>0.90 Tinsley, Adamson et al. (2020) [41]	−2.66 (<0.001) Cabre et al. (2021) [36]<br>−3.1 Tinsley, Adamson et al (2020) [34]<br>−0.3 Tinsley, Adamson et al (2020) [34]<br>−0.5 Tinsley, Adamson et al (2020) [34]<br>−0.1 Tinsley, Adamson et al (2020) [34]	−4.47, 9.79 Cabre et al. (2021) [36]<br>−9.8, 3.6 Tinsley, Adamson et al. (2020) [41]<br>−7.3, 6.7 Tinsley, Adamson et al. (2020) [41]<br>−5.8, 4.8 Tinsley, Adamson et al. (2020) [41]<br>−7.3, 7.1 Tinsley, Adamson et al. (2020) [41]
Total fat free mass	DXA	Lean mass: 0.9 Cabre et al. (2021) [36]	0.98 Wong et al. (2019) [37]<br>Lean mass: 0.88 Cabre et al. (2021) [36]<br>0.85 Ng et al. (2016) [45]		1.83 Wong et al. (2019) [37]<br>Lean mass: 3.77 Cabre et al. (2021) [36]<br>3.14 Ng et al. (2016) [45]		Lean mass: 0.1 (0.71) Cabre et al. (2021) [36]	Lean mass: −7.68, 7.48 Cabre et al. (2021) [36]
	4C model	0.92 Cabre et al. (2021) [36]	0.84 Cabre et al. (2021) [36]	4.76 Cabre et al. (2021) [36]	4.6 Tinsley, Adamson et al. (2020) [41]<br>3.6 Tinsley, Adamson et al. (2020) [41]<br>2.8 Tinsley, Adamson et al. (2020) [41]<br>3.7 Tinsley, Adamson et al. (2020) [41]	0.93 Tinsley, Adamson et al. (2020) [41]<br>0.96 Tinsley, Adamson et al. (2020) [41]<br>0.97 Tinsley, Adamson et al. (2020) [41]<br>0.95 Tinsley, Adamson et al. (2020) [41]	3.15 (<0.001) Cabre et al. (2021) [36]<br>3.1 Tinsley, Adamson et al. (2020) [41]<br>0.3 Tinsley, Adamson et al. (2020) [41]<br>0.5 Tinsley, Adamson et al. (2020) [41]<br>0.1 Tinsley, Adamson et al. (2020) [41]	−14.9, 3.78 Cabre et al. (2021) [36]<br>−3.6, 9.8 Tinsley, Adamson et al. (2020) [41]<br>−6.7, 7.3 Tinsley, Adamson et al. (2020) [41]<br>−4.9, 5.9 Tinsley, Adamson et al. (2020) [41]

							−7.1, 7.3 Tinsley, Adamson et al. (2020) [41]
Visceral fat mass	DXA		0.92 Wong et al. (2019) [37]		0.15 Wong et al. (2019) [37]		
Body volume	DXA		0.98 Sobhiyeh, Dunkel et al. (2021) [40]<br>1.0 Sobhiyeh, Dunkel et al. (2021) [40]<br>0.974 Ng et al. (2016) [45]		2.73 Ng et al. (2016) [45]	−11.64 Sobhiyeh, Dunkel et al. (2021) [40]<br>−1.3 Sobhiyeh, Dunkel et al. (2021) [40]	
	ADP		0.99 Bourgeois et al. (2017) [34]<br>0.99 Bourgeois et al. (2017) [34]<br>0.99 Bourgeois et al. (2017) [34]<br>0.98 Sobhiyeh, Dunkel et al. (2021) [40]<br>1.00 Sobhiyeh, Dunkel et al. (2021) [40]		4.2 Bourgeois et al. (2017) [34]<br>2.9 Bourgeois et al. (2017) [34]<br>9.7 Bourgeois et al. (2017) [34]	−3.9 (041) Tinsley, Benavides et al (2020) [35]<br>8.0 (<0.001) Tinsley, Benavides et al (2020) [35]<br>−10.1 (<0.001) Tinsley, Benavides et al (2020) [35]<br>−9.58 Sobhiyeh, Dunkel et al. (2021) [40]<br>5.24 Sobhiyeh, Dunkel et al. (2021) [40]	
	UHWW	0.99 Pepper et al. (2010) [31]	0.99 Pepper et al. (2010) [31]<br>0.99 Wang et al. (2006) [46]	1.6 Pepper et al. (2010) [31]<br>0.892 Wang et al. (2006) [46]		0.2 (0.3) Pepper et al. (2010) [31]<br>−0.46 (<0.001) Wang et al. (2006) [46]	2.92, 3.32 Pepper et al. (2010) [31]
Trunk volume	DXA		0.97 Tinsley, Benavides et al. (2020) [47]		9.2 Tinsley, Benavides et al. (2020) [47]	8.8 (<0.001) Tinsley, Benavides et al. (2020) [47]	2.2, 15.4 Tinsley, Benavides et al. (2020) [47]

		0.97 Tinsley, Benavides et al. (2020) [47]<br>0.96 Tinsley, Benavides et al. (2020) [47]<br>0.97 Sobhiyeh, Dunkel et al. (2021) [40]<br>0.98 Sobhiyeh, Dunkel et al. (2021) [40]<br>0.974 Ng et al. (2016) [45]<br>0.97 Bourgeois et al. (2017) [34]<br>0.97 Bourgeois et al. (2017) [34]<br>0.98 Bourgeois et al. (2017) [34]	15.1 Tinsley, Benavides et al. (2020) [47]<br>3.6 Tinsley, Benavides et al. (2020) [47]<br>1.95 Ng et al. (2016) [45]<br>10.7 Bourgeois et al. (2017) [34]<br>14.0 Bourgeois et al. (2017) [34]<br>6.4 Bourgeois et al. (2017) [34]	14.8 (<0.001) Tinsley, Benavides et al. (2020) [47]<br>3.2 (0.02) Tinsley, Benavides et al. (2020) [47]<br>−0.47 Sobhiyeh, Dunkel et al. (2021) [40]<br>5.6 Sobhiyeh, Dunkel et al. (2021) [40]	7.7, 21.9 Tinsley, Benavides et al .(2020) [35]<br>−0.6, 7.0 Tinsley, Benavides et al. (2020) [47]
Right arm volume	DXA	0.93 Tinsley, Benavides et al. (2020) [47]<br>0.67 Tinsley, Benavides et al. (2020) [47]<br>0.87 Tinsley, Benavides et al. (2020) [47]<br>0.75 Sobhiyeh, Dunkel et al. (2021) [40]	3.0 Tinsley, Benavides et al (2020) [35]<br>1.6 Tinsley, Benavides et al (2020) [35]<br>5.7 Tinsley, Benavides et al (2020) [35]<br>0.57 Ng et al. (2016) [45]<br>0.9 Bourgeois et al. (2017) [34]	−2.9 (<0.001) Tinsley, Benavides et al (2020) [35]<br>−0.9 (<0.001) Tinsley, Benavides et al (2020) [35]<br>−5.6 (<0.001) Tinsley, Benavides et al (2020) [35]<br>−2.08 Sobhiyeh, Dunkel et al. (2021) [40]<br>−1.05 Sobhiyeh, Dunkel et al. (2021) [40]	−10.8, 5.0 Tinsley, Benavides et al. (2020) [47]<br>−7.1, 5.3 Tinsley, Benavides et al. (2020) [47]<br>−13.0, 1.8 Tinsley, Benavides et al. (2020) [47]

		0.79 Sobhiyeh, Dunkel et al. (2021) [40]<br>0.87 Ng et al. (2016) [45]<br>0.87 Bourgeois et al. (2017) [34]<br>0.89 Bourgeois et al. (2017) [34]<br>0.80 Bourgeois et al. (2017) [34]	1.1 Bourgeois et al. (2017) [34]<br>2.5 Bourgeois et al. (2017) [34]		
Left arm volume	DXA	0.93 Tinsley, Benavides et al. (2020) [47]<br>0.67 Tinsley, Benavides et al. (2020) [47]<br>0.87 Tinsley, Benavides et al. (2020) [47]<br>0.75 Sobhiyeh, Dunkel et al. (2021) [40]<br>0.79 Sobhiyeh, Dunkel et al. (2021) [40]<br>0.87 Ng et al. (2016) [45]<br>0.82 Bourgeois et al. (2017) [34]<br>0.83 Bourgeois et al. (2017) [34]<br>0.69 Bourgeois et al. (2017) [34]	3.0 Tinsley, Benavides et al. (2020) [47]<br>1.6 Tinsley, Benavides et al. (2020) [47]<br>5.7 Tinsley, Benavides et al. (2020) [47]<br>0.57 Ng et al. (2016) [45]<br>0.9 Bourgeois et al. (2017) [34]<br>0.8 Bourgeois et al. (2017) [34]<br>2.4 Bourgeois et al. (2017) [34]	−2.9 (<0.001) Tinsley, Benavides et al (2020) [35]<br>−0.9 (<0.001) Tinsley, Benavides et al (2020) [35]<br>−5.6 (<0.001) Tinsley, Benavides et al (2020) [35]<br>−2.08 Sobhiyeh, Dunkel et al. (2021) [40]<br>−1.05 Sobhiyeh, Dunkel et al. (2021) [40]	−10.8, 5.0 Tinsley, Benavides et al. (2020) [47]<br>−7.1, 5.3 Tinsley, Benavides et al. (2020) [47]<br>−13.0, 1.8 Tinsley, Benavides et al. (2020) [47]

Right leg volume	DXA	0.65 Tinsley, Benavides et al. (2020) [47] 0.76 Tinsley, Benavides et al. (2020) [47] 0.82 Tinsley, Benavides et al. (2020) [47] 0.86 Sobhiyeh, Dunkel et al. (2021) [40] 0.89 Sobhiyeh, Dunkel et al. (2021) [40] 0.73 Ng et al. (2016) [45] 0.90 Bourgeois et al. (2017) [34] 0.74 Bourgeois et al. (2017) [34] 0.91 Bourgeois et al. (2017) [34]	10.0 Tinsley, Benavides et al. (2020) [47] 6.2 Tinsley, Benavides et al. (2020) [47] 14.8 Tinsley, Benavides et al. (2020) [47] 1.546 Ng et al. (2016) [45] 3.7 Bourgeois et al. (2017) [34] 5.0 Bourgeois et al. (2017) [34] 7.1 Bourgeois et al. (2017) [34]	−9.1 (<0.001) Tinsley, Benavides et al. (2020) [47] −5.3 (<0.001) Tinsley, Benavides et al. (2020) [47] −14.3 (<0.001) Tinsley, Benavides et al. (2020) [47] −3.77 Sobhiyeh, Dunkel et al. (2021) [40] −1.59 Sobhiyeh, Dunkel et al. (2021) [40]	−10.5, −7.7 Tinsley, Benavides et al. (2020) [47] −7.9, −2.7 Tinsley, Benavides et al. (2020) [47] −16.6, −12.0 Tinsley, Benavides et al. (2020) [47]
Left leg volume	DXA	0.65 Tinsley, Benavides et al. (2020) [47] 0.76 Tinsley, Benavides et al. (2020) [47] 0.82 Tinsley, Benavides et al. (2020) [47]	10.0 Tinsley, Benavides et al. (2020) [47] 6.2 Tinsley, Benavides et al. (2020) [47] 14.8 Tinsley, Benavides et al. (2020) [47] 1.546 Ng et al. (2016) [45]	−9.1 (<0.001) Tinsley, Benavides et al. (2020) [47] −5.3 (<0.001) Tinsley, Benavides et al. (2020) [47] −14.3 (<0.001) Tinsley, Benavides et al. (2020) [47] −3.77 Sobhiyeh, Dunkel et al. (2021) [40]	−10.5, −7.7 Tinsley, Benavides et al. (2020) [47] −7.9, −2.7 Tinsley, Benavides et al. (2020) [47] −16.6, −12.0 Tinsley, Benavides et al. (2020) [47]

	0.86 Sobhiyeh, Dunkel et al. (2021) [40]<br>0.89 Sobhiyeh, Dunkel et al. (2021) [40]<br>0.73 Ng et al. (2016) [45]<br>0.9 Bourgeois et al. (2017) [34]<br>0.7 Bourgeois et al. (2017) [34]<br>0.91 Bourgeois et al. (2017) [34]	3.9 Bourgeois et al. (2017) [34]<br>4.8 Bourgeois et al. (2017) [34]<br>7.0 Bourgeois et al. (2017) [34]	−1.59 Sobhiyeh, Dunkel et al. (2021) [40]

* When multiple values of the same accuracy index (e.g., R^2) are displayed for the same study, each value refers to a different digital scanner (commercial name not reported). Abbreviations: BIA = bioimpedance analysis; DXA = dual-energy X-ray absorptiometry; ADP = air displacement plethysmography; UHWW = hydrostatic weighing.
